# BioShield-12: A 3D-Printed Conformal Chest Shield for Wireless 12-Lead ECG Acquisition

**DOI:** 10.3390/s26154936

**Published:** 2026-08-04

**Authors:** Ahsan Naveed, Rida-e-Fatima, Zia Mohy Ud Din, Abdullah Al Aishan, Hedi Ammar Guesmi, Jahan Zeb Gul

**Affiliations:** 1 Department of Biomedical Engineering, Air University, Islamabad 44000, Pakistan; ahsan.naveed@au.edu.pk (A.N.); ridafatima1401@gmail.com (R.-e.-F.); drzia@au.edu.pk (Z.M.U.D.); 2 Department of Basic Medical Sciences, College of Applied Medical Sciences, King Khalid University, Abha 61421, Saudi Arabia; aalaishan@kku.edu.sa; 3 Department of Electrical Engineering, College of Engineering, Qassim University, Buraydah 52571, Saudi Arabia; 4 Department of Electronic Engineering, Maynooth University, W23 A3HY Maynooth, Ireland; jahanzeb.gul@mu.ie

**Keywords:** additive manufacturing, 3D printing, wearable ECG, chest shield, electrode placement, fused deposition modeling

## Abstract

Reproducible electrode placement remains a critical, unresolved challenge in wearable ECG systems, where manual electrode attachment introduces inter-session positional error that degrades signal morphology and compromises multi-lead representation. Although the 12-lead clinical ECG system is the gold standard, conventional setups are often bulky, wired, and dependent on operator expertise, limiting their use in prehospital or remote care. Recent advancements in wearable and wireless ECG systems have improved mobility and real-time monitoring, but they typically suffer from limited lead coverage, discomfort, and unstable connectivity. This paper introduces BioShield-12, an anatomically adaptive thermoplastic polyurethane (TPU) shield fabricated using fused deposition modeling (FDM) to improve multi-site electrode placement and 12-lead electrocardiogram (ECG) reconstruction from a single-shield design based on anthropometric data from ten healthy adults (five male, five female; sizing cohort). Mechanical characterization confirmed TPU’s suitability as a compliant wearable substrate, with toughness of 34.4 MJ/m^3^ and elastic modulus of 79.1 MPa. Feasibility validation against a research-grade reference (BIOPAC MP36) in twenty healthy subjects (n = 20; 10 male, 10 female; mean age 21 ± 4 years) demonstrated consistent signal fidelity (SNR: 17.5–22.5 dB; Pearson r: 0.93–0.98; power-line interference ratio (PLIR): 1.0–3.2 × 10^−5^) and confirmed feasibility of 12-lead reconstruction. This work provides proof-of-concept for an additive manufacturing-based conformal electrode interface that eliminates variability in placement without needing individual attachment.

## 1. Introduction

Accurate and repeatable electrode placement is a basic, well-recognized problem in wearable electrocardiography (ECG) systems, especially in inpatient and ambulatory environments where the skills of the operator cannot always be assured. Ten electrodes are required to be placed accurately on the limbs and chest for the standard 12-lead ECG, and even slight variation in the placement of the electrodes from the recommended anatomical locations introduces inter-session variability, which then affects the ECG waveform, diagnostic intervals, and the ability to compare longitudinal ECGs; hence the need for trained professionals to place electrodes. The clinical consequences of placement error are non-trivial: precordial electrode displacement of as little as 2 cm has been shown to alter QRS amplitude and ST-segment contour sufficiently to affect automated interpretation [1]. In prehospital and resource-limited environments, where trained personnel may not be available to supervise each electrode attachment, this inconsistency is compounded [2]. A wearable system in which the electrodes are fixed in a geometrically predetermined pattern would alleviate this issue, enabling a nurse or first responder to record a 12-lead ECG without manual placement of each electrode. While there has been a rapid growth in the field of wearable ECGs in the last ten years [3], recent state-of-the-art platforms have extended this trend toward continuous wearable 12-lead acquisition coupled with large-scale machine-learning interpretation [4], underscoring that the dominant research direction has shifted from hardware connectivity alone toward standardized, analysis-ready multi-lead data. Nevertheless, current systems continue to compromise between the number of leads, the reproducibility of placement, comfort and ease of setup, with no reported device meeting all four criteria.

A review of existing commercial and research-grade wearable ECG systems reveals a recurring gap between lead coverage and placement standardization. Flexible electrode systems [5] and textile-based sensors [6] provide comfort and conformability but rely on manual placement that varies between sessions and operators. Multi-lead wearable platforms, such as vest-based systems [7], improve coverage but require individual electrode attachment and remain bulky. Recent reviews of dry-electrode ECG sensing similarly conclude that, while electrode materials and skin-contact chemistry have advanced substantially, reproducible electrode positioning remains an unsolved and under-addressed bottleneck for unsupervised wearable use [8]. Microcontroller-based telemetry designs have improved wireless capability but typically offer limited lead counts [9], and even those achieving 3–6 leads do not address the underlying problem of placement reproducibility. Full 12-lead wearable acquisition remains technically challenging because it requires simultaneous multi-channel acquisition, consistent electrode geometry across sessions and body shapes, and mechanical stability during motion.

KardiaMobile 6L (AliveCor, Inc.) sourced from Mountain View, California, United States [9] is an FDA-cleared, 6-lead handheld ECG device widely adopted for atrial fibrillation screening; however, its non-wearable contact-based format requires the user to hold the device for a 30 s snapshot acquisition, precluding continuous monitoring and providing only 6 of the standard 12 leads, limiting utility in emergency scenarios where a wearable, hands-free format is preferred. Vest-based multi-lead systems (e.g., SmartVest, Zoll LifeVest) [10] integrate textile electrodes into a wearable garment and can support up to 12-lead coverage, but their bulk, washability requirements, and high cost ($500–$3000) limit deployment in resource-constrained or emergency prehospital settings. Wang et al. proposed a low-power multi-lead wearable ECG system with on-board sensor data compression achieving three leads [11]; while their approach excels in power efficiency, it does not provide simultaneous full 12-lead acquisition. Sriraam et al. [12] demonstrated a single-lead flexible textile sensor for continuous monitoring with acceptable signal quality, but single-lead systems cannot replicate the spatial multi-vector information needed for comprehensive 12-lead reconstruction [13]. A complementary body of recent work attacks this same coverage gap purely in software, using deep learning to synthesize the absent leads from a reduced electrode set—for example, wavelet-domain long short-term memory networks that exploit inter- and intra-lead correlation [14] and transformer-based reconstruction from EASI or Frank leads with clinical validation [15]. These data-driven methods can achieve high waveform correlation, but they infer unmeasured potentials from population-learned priors rather than acquiring them, which limits patient-specific amplitude fidelity and introduces a model-dependent failure mode. BioShield-12 instead resolves the coverage gap at the acquisition layer: it physically constrains where every electrode lands and derives the dependent leads through closed-form, patient-independent identities, so the reconstruction is deterministic and traceable rather than statistically estimated. Table 1 contrasts the key design axes of representative wearable ECG platforms with BioShield-12. Among the platforms reviewed, none simultaneously combines full 12-lead coverage, geometric placement standardization, and single-piece wearability in an integrated interface. BioShield-12 is designed to address this combination by encoding electrode locations as fixed geometric features of a conformal 3D-printed carrier and reconstructing the 12 leads from 11 manubrium-referenced acquisition sites.

The wearable healthcare industry has taken a recent interest in new developments in 3D printing that offer possibilities of manufacturing not easily achieved through traditional production methods. Researchers have used 3D scans of the body to print wearable devices that conform to the surface of body parts such as the torso, with sensor placement embedded into the printed structure to facilitate physiological measurements that cannot be made with non-conformal devices [16]. The state of the art in additively manufactured bioelectronics has advanced rapidly: micro-architected printed electrodes now enlarge true skin-contact area to lower interface impedance [17], and direct-ink-write conductive composites yield conformable ECG patches that sustain high-fidelity recording over weeks of wear [18]. This work demonstrates that additive manufacturing is being used not merely to package electronics but to engineer the electrode–skin interface itself. Building on this structural capability, a chest-worn cardiorespiratory monitoring system has been reported in which integrated conductive sensors and an IMU are housed inside a custom 3D-printed TPU casing placed directly on the chest wall [19]. Despite this progress, existing wearable ECG interfaces remain constrained by the limitations of their underlying platforms. Adhesive patch systems typically support only one to three leads, while textile vest platforms require per-session individual electrode attachment [6,11] and remain susceptible to positional drift during thoracic motion. Till now, there has been no reported conformally structured wearable system that both geometrically constrains electrode placement across the anterior thorax and inter-electrode distance (without user intervention) and enables 12-lead ECG reconstruction. This calls for an interface that connects the structural design freedoms afforded by 3D printing with the need for consistent multi-lead cardiac measurements.

**Table 1:** Comparison of representative wearable and portable ECG platforms against BioShield-12. Columns reflect the design axes most relevant to multi-lead wearable acquisition: reported lead count, whether electrode placement is standardized by the device geometry, electrode type (dry vs. wet/noncontact), and whether the 12-lead ECG is directly acquired or reconstructed. All cited literature entries are peer-reviewed publications. The BioShield-12 row is based on in-house prototype testing.

System	Lead Count	Placement Standardized	Electrode Type	12-Lead Recon.	Per-Session Placement Required
KardiaMobile 6L [9]	6	No (handheld)	Dry metal	No	Yes
Hsu et al.	12	No (manually adjustable)	Noncontact (capacitive)	Yes (direct)	Yes
Wang et al. [11]	3	No (manual on vest)	Dry (silicone)	No	Yes
Sriraam et al. [12]	1	No	Dry textile (Ag-NyW)	No	Yes
Di Tocco et al. [19]	Cardiorespiratory	Partial	Conductive textile	No	Partial
Stuart et al. [16]	ECG + BioZ	No (body-scan-derived)	FDM dry	Yes	Yes (per-person scan)
BioShield-12	12	Yes (geometric encoding)	Dry Ag/AgCl	Yes	No

In this paper, BioShield-12 is presented, which is a 3D-printed anatomically compliant thermoplastic polyurethane (TPU) chest shield that enforces standardized multi-site electrode placement without operator intervention by conforming directly to the thorax. This additive manufacturing-enabled architecture embeds lead geometry as a structural property of the device rather than a procedural step dependent on clinical skill. Built upon this foundation, a compact PCB enclosure housing the acquisition electronics enables wireless 12-lead ECG reconstruction in a self-contained, reproducible wearable system.

## 2. Materials and Methods

**Figure 1:**
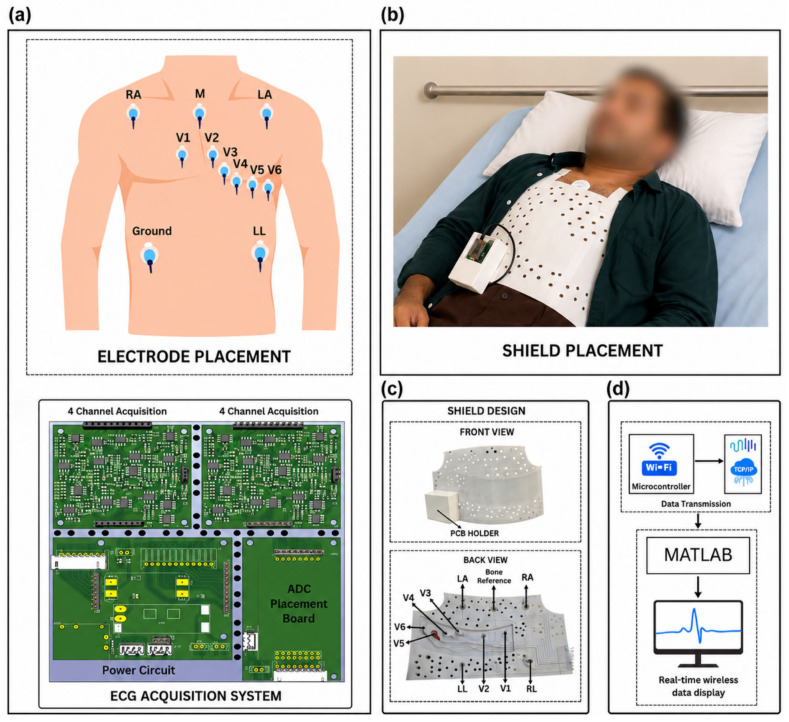
Architecture of the BioShield-12 12-lead ECG acquisition system. (**a**) Schematic electrode placement: precordial (V1–V6) and torso limb positions (RA, LA, LL) with the two 4-channel acquisition units, ADC board, and power circuit. (**b**) Embedded electrode-channeled wearable TPU shield, placed over the thorax at the time of recording. (**c**) The front and back of the shield depict the process of electrode integration, and a sheer PCB enclosure ensures constant contact of the electrodes with the skin, minimizing the effects of movement artifacts. (**d**) Wireless transmission of data from the ESP32 S3 microcontroller to the MATLAB client to visualize and analyze data in real time.

The overall methodology of BioShield-12 comprises a geometrically standardized, anatomically conformal TPU carrier of electrode channels, a custom-designed multi-channel ECG analog front-end (AFE), and a wireless telemetry subsystem to stream real-time data, as shown in Figure 1. The structural base of the system is a 3D-printed TPU chest shield that has standard electrode locations, including precordial (V1–V6) and torso limb positions (RA, LA, LL), which are encoded as fixed spatial features and no longer need to be placed manually on a per-session basis, as presented in Figure 1a. The shield is worn directly over the thorax, as shown in Figure 1b, and was printed using fused deposition modeling (FDM) and mechanically characterized to ensure it was an appropriate compliant wearable substrate. The front and back of the shield illustrate the electrode integration process, with a PCB enclosure maintaining consistent electrode–skin contact to minimize motion artifacts (Figure 1c). The shield enclosure contains a custom 8-channel ECG acquisition PCB and a wireless telemetry subsystem that transmits signals in real time via the ESP32 S3 microcontroller to a MATLAB R2024a client for downstream visualization and analysis (Figure 1d).

### 2.1. Wearable Shield Design and Fabrication

In resource-limited and prehospital environments, the traditional manual positioning of electrodes creates operator dependency, which is especially challenging for achieving reproducible multi-site ECG acquisition. To overcome this, a sizing cohort produced anthropometric measurements that were then used to produce a one-size conformal shield footprint with inbuilt fixed electrode locations, routed channels, and strain-relief paths etched into the printed substrate. A front-side electronics enclosure ensured mechanical stability at the electrode–skin interface while removing the cable overhead of conventional Holter-like systems [20]. The design and 3D printing of the shield are discussed in detail in the succeeding subsections.

#### 2.1.1. 3D Modeling and Printing of the Shield

**Figure 2:**
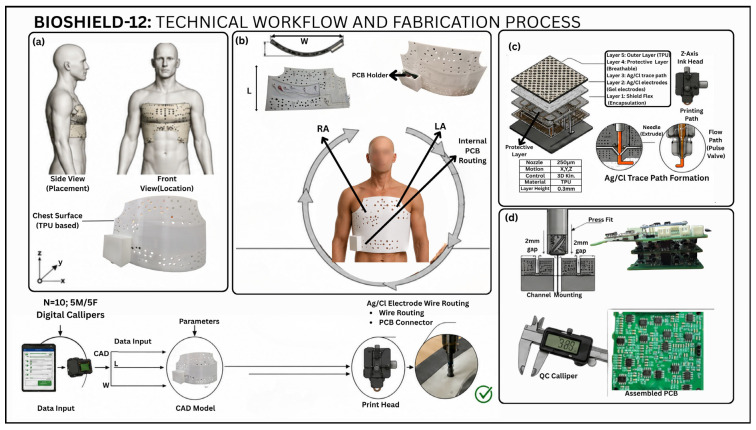
BioShield-12 additive manufacturing fabrication workflow: (**a**) anthropometric data collection from n = 10 volunteers (5M/5F) using digital calipers at anatomical landmarks to parameterize electrode locations; (**b**) parametric conformal CAD modeling of the TPU shield with 11 electrode channels (ten measurement sites plus one manubrium reference) and integrated PCB holder; (**c**) precision FDM printing on a Bambu Lab X1C using TPU 95A filament at 220 °C nozzle temperature, 0.2 mm layer height, gyroid 40% infill at 40 mm/s; (**d**) post-print quality control including channel reaming to 10 mm and dimensional verification with calipers.

Anthropometric landmarks for CAD modeling of the shield were measured on 10 healthy adult volunteers (5 male, 5 female; sizing cohort) using digital calipers at anatomical landmarks as shown in Figure 2a. Measurements recorded per volunteer included anterior chest width (clavicle-to-clavicle), sternum length (manubrium to xiphoid process), and intercostal heights of the standard precordial electrode sites (V1–V6) relative to the sternal angle. Torso-repositioned limb electrode sites (RA, LA, LL) were referenced to the infraclavicular fossa and the inferior costal margin, respectively [13]. Electrode-site centroids were encoded as dimensionally fixed features in the CAD model (recessed channels of 10 mm diameter, 2 mm depth) relative to the manubrium as a stable anatomical reference point, so the printed geometry enforces inter-electrode spacing without operator intervention. The shield periphery incorporates adjustable retention ends, allowing the fit to be tightened or loosened across a range of chest widths to accommodate intra-individual dimensional variation that arises from typical body weight fluctuation without requiring a re-print. Three functional sub-regions were defined in the CAD model as shown in Figure 2b: (i) the electrode carrier surface comprising eleven recessed electrode channels (ten measurement sites V1–V6, RA, LA, LL, RL and one manubrium reference); (ii) strain-relief cable-routing channels (3 mm wide, 1.5 mm deep) running from each electrode site to the central PCB enclosure; and (iii) a front-face PCB enclosure housing the 8-channel acquisition electronics, from which the 12 standard leads are reconstructed (Section Channel-to-Lead Mapping: From 8 Acquisition Channels to 12 Reconstructed Leads). Print parameters were layer height 0.2 mm, nozzle temperature 220 °C, bed temperature 35 °C, print speed 40 mm/s, gyroid infill at 40% density, three perimeter walls, and four top/bottom layers. These parameters were selected after pilot prints to balance mechanical compliance, dimensional accuracy, and print reliability with TPU, as shown in Figure 2c. Following printing, post-print quality control was performed on the single prototype shield used throughout this study, including channel reaming to 10 mm and dimensional verification with calipers, as shown in Figure 2d, after which final electrode mounting and assembly with Ag/AgCl contacts, internal wire routing, and PCB integration were completed.

#### 2.1.2. Material Characterization and Selection

**Figure 3:**
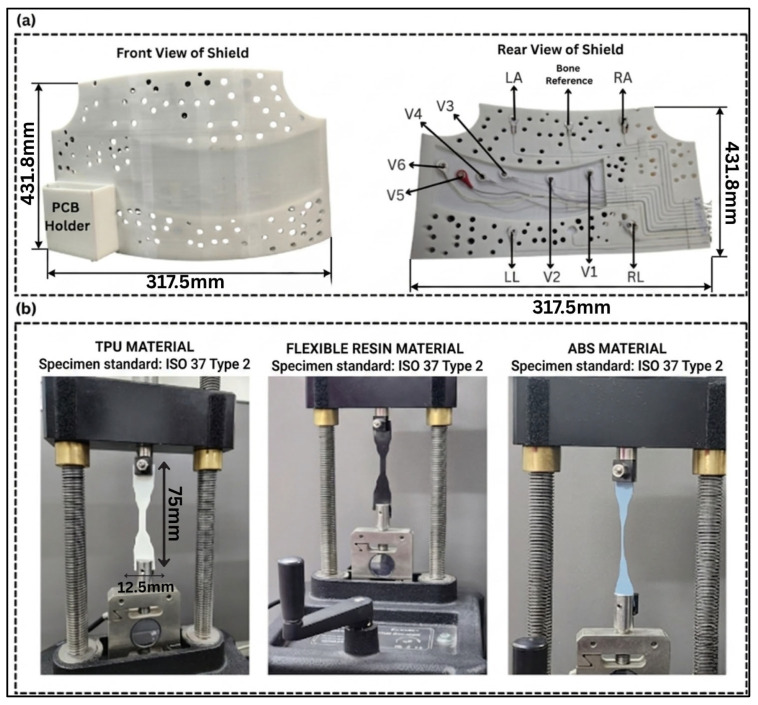
(**a**) CAD rendering and 3D-printed prototype of the BioShield-12 chest shield depicting anatomically guided electrode positions. (**b**) PASCO mechanical test system that was utilized to test the tensile strength of TPU, ABS, and flexible resin samples.

The appropriate material selection for our shield was guided by the mechanical functional requirements of the wearable substrate [21]. The mechanically compliant components required a material that provides flexibility [22] and user comfort while also offering favorable sustainability characteristics relative to single-use adhesive patches, including material reusability and reduced per-session consumable waste [23]. Therefore, three candidate materials, including thermoplastic polyurethane (TPU), acrylonitrile butadiene styrene (ABS), and a flexible photopolymer resin, were selected to span a broad stiffness–ductility envelope for comparative tensile evaluation. Uniaxial tensile testing was conducted on standardized specimens (prescribed gauge length and cross-sectional area) for each candidate material under monotonic loading at a constant crosshead displacement rate until fracture. A PASCO Materials Testing Machine (ME 8236) that has a 7100 N load cell and an optical displacement encoder was used to carry out the experiments to measure force and displacement at high resolution. Force–displacement data were acquired via PASCO Capstone software and converted to engineering stress–strain curves for analysis. From these curves, Young’s modulus (E), ultimate tensile strength (UTS), and toughness (area under the stress–strain curve to fracture) were derived for each material as presented in Figure 3b, along with the final printed TPU shield shown in Figure 3a.

#### 2.1.3. Shield Fabrication

Fused Deposition Modeling (FDM) on a desktop-level printer was employed for the fabrication of the BioShield-12 TPU chest shield. Based on its Shore hardness of 95A [24], thermoplastic polyurethane (TPU 95A) was chosen as the print material due to its ability to offer the necessary structural rigidity to maintain electrode press-fit geometry, but to be compliant enough to follow thoracic curvature without the use of rigid fixation hardware. The nozzle temperature of 220 °C, bed temperature of 35 °C, and print speed of 40 mm/s were consistent with the manufacturer’s recommended processing window for 95A Shore TPU filament and aligned with established FDM process design principles governing liquefier dynamics, bead spreading, and build surface adhesion [25]. The parameters, as shown in Table 2, were finally set to use a layer height of 0.20 mm, an infill density of 40% (gyroid pattern), and three perimeter walls. The gyroid infill topology [26] was chosen over rectilinear alternatives because it distributes compressive load isotropically across all axes, lowering stress concentrations at the edges of the electrode channels under repeated mechanical cycling. To keep thermal gradients between layers to a minimum, the cooling fan speed was limited to 30%. Elevated interlayer thermal gradients are recognized as a contributor to interlayer delamination in flexible filament printing [25]. It is acknowledged that FDM printing of flexible filaments introduces process-level variability across print runs, including dimensional tolerances arising from thermal expansion and nozzle-filament interaction, infill density variation due to retraction and stringing behavior characteristic of TPU, and interlayer adhesion inconsistency driven by thermal gradients between deposited layers. In BioShield-12, three design and process decisions were taken to minimize the impact of these sources of variability on signal and wear consistency. First, the post-print channel reaming step (Section 2.1.4) decouples final electrode channel diameter from as-printed dimensional accuracy, ensuring press-fit electrode seating is governed by the reaming tool geometry (10.0 mm ± 0.05 mm) rather than by raw print fidelity. Second, the gyroid infill topology distributes compressive load isotropically, making electrode contact pressure less sensitive to local infill density variation than rectilinear alternatives would be. Third, the 30% cooling fan speed limit reduces interlayer thermal gradients, mitigating the primary driver of interlayer delamination in TPU FDM parts [25]. These measures reduce but do not eliminate print-to-print variability; multi-unit manufacturing reproducibility has not been characterized in the present work and is identified as a required step before deployment beyond the current single-prototype feasibility demonstration.

#### 2.1.4. Post-Processing Workflow

**Figure 4:**
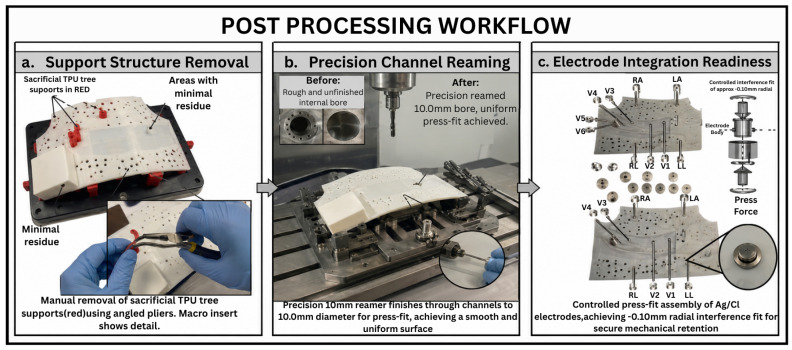
Once the TPU shield is printed, (**a**) tree support structures are to be cleared out by hand. (**b**) Electrode channel reaming, dimensional accuracy, and press-fit compatibility of electrodes. (**c**) The Ag/AgCl dry electrode is inserted into the placeholder by pressing.

After fabrication, three stages of systematic post-processing were performed on each BioShield-12 prototype to ensure dimensional accuracy, surface quality, and electrode integration readiness. Sacrificial TPU tree supports were first removed manually at the first stage with angled needle-nose pliers, with care taken to leave minimum surface residue as shown in Figure 4a. The second step was precision channel reaming, whereby a high-speed 10 mm steel fluted reamer finished the through-channels to a final bore diameter of 10.0 mm (within a tolerance of 0.05 mm), changing the bore finish to a smooth and uniform surface suitable for press-fit assembly, as depicted in Figure 4b. Lastly, on the third stage, Ag/AgCl dry electrodes were assembled through a controlled press-fit mechanism, which provided a tight mechanical fixation as shown in Figure 4c. Contact pressure at the electrode–skin interface and skin–electrode impedance (SEI) were not quantified in the present study, as BioShield-12 is a first-generation feasibility prototype; the gyroid infill topology and press-fit channel geometry were selected to provide passive, repeatable mechanical seating, with signal quality metrics (SNR, Pearson r, PLIR) serving as indirect indicators of interface adequacy. Each step of this workflow is shown in Figure 4. Print process repeatability across manufactured units, specifically, dimensional tolerances across electrode channels, infill density variation, and interlayer adhesion consistency, represents a key unknown that must be quantified before signal and wear consistency can be assured beyond the current single-prototype scope. A systematic multi-unit print study characterizing electrode channel diameter tolerance, shield footprint dimensional deviation, and mechanical property consistency across a minimum production batch is identified as immediate future work preceding any clinical deployment consideration.

#### 2.1.5. Placement Reliability

**Figure 5:**
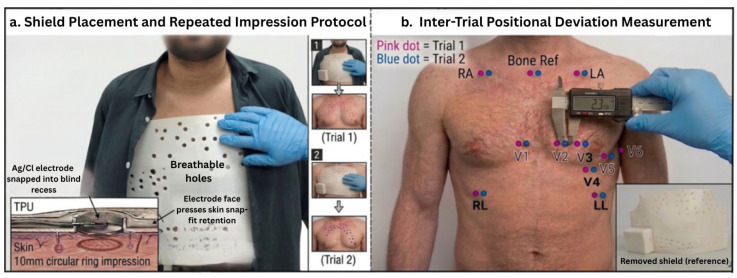
Electrode placement validation procedure: (**a**) BioShield-12 pressed against the anterior chest, allowing blind electrode recesses to imprint 10 mm circular marks on skin, and (**b**) post-removal inter-electrode distances measured with a Vernier caliper (resolution: 0.02 mm) across all 11 sites.

The placement reliability of BioShield-12 was tested in the framework of a repeated impression protocol that was performed on 5 healthy adults, as shown in Figure 5a. The shield was firmly placed on the anterior chest wall, and each of the blind electrode recesses left a circular mark on the skin at all 11 electrode locations (V1–V6, RA, LA, LL, RL, and the manubrial reference). After removal, a surgical skin marker was immediately dotted on the impressions. This was done 3 times on each subject, and the shield was completely taken off and reattached between each trial as depicted in Figure 5b. Each of the 3 distinct trial pairings per subject (T1-T2, T1-T3, and T2-T3) was assessed, resulting in 165 total inter-trial deviation measures (5 subjects, 3 pairs, 11 sites). The straight-line distance between the two impression dots of a given trial pair at each electrode site was measured with a Vernier caliper, which is the positional deviation caused by the removal and reapplication of shields. The mean deviation and standard deviation were calculated on a per-electrode-site basis and an aggregate basis on all measurements.

### 2.2. ECG Acquisition System and Signal Validation

Since electrocardiography signals are low-amplitude cardiac biopotentials (usually 0.5–5 mV on the body surface), they are susceptible to baseline drift, power-line interference, and motion artifact; the AFE should therefore have a high common-mode rejection ratio (CMRR), full diagnostic ECG bandwidth (0.05–100 Hz), and sufficient gain to fill the ADC dynamic range [27,28]. The nominal front-end voltage gain of 800 *v*/*v* is split into 8 *v*/*v* at the INA129 instrumentation amplifier stage, then another gain stage of 100 *v*/*v* at the post-filter stage. The AFE architecture that was adopted (the entire schematic is provided in the Supplementary Section S1) is a four-stage cascaded signal-conditioning chain [29]. In the first stage, an INA129 instrumentation amplifier [30] is used, chosen because of its high input impedance (≥10 GΩ) and common-mode rejection ratio (CMRR ≥ 120 dB), which together reduce the effects of electrode polarization and common-mode interference at the electrode–skin interface.

A pre-amplifier is followed by an active Twin-T 50 Hz notch filter to remove power-line interference. This is followed by a high-pass filter (0.01 Hz) which eliminates the DC offset and low-frequency baseline drift caused by respiration and motion while not modifying the ST segment and the T-wave. A final gain stage (100 *v*/*v*) is used to bring the ECG signal to the full dynamic range of the ADC. A 100 Hz low-pass stage sets the diagnostic bandwidth and acts as the anti-aliasing filter prior to digitization. Digitization is performed by a 24-bit delta-sigma ADC (ADS1256) [28] configured in differential mode. The ADS1256 has no built-in biopotential features such as right-leg drive or lead-off detection. The multi-stage analog conditioning chain removes noise ahead of the converter, and the cascaded gain architecture ensures that the conditioned ECG occupies a large proportion of the ADC dynamic range, rendering input-referred quantization noise insignificant relative to the signal amplitude. The single-ended reference for the acquisition channels is provided by a manubrium reference electrode tied to the analog ground plane, which also rejects common-mode interference. The effectiveness of this chain is reflected experimentally in the measured power-line interference ratio (PLIR ≈ 1.0–3.2 × $10^{-5}$) of the recorded waveforms. The system-level common-mode rejection ratio (CMRR) was evaluated following the computational framework from the measured residual 50 Hz amplitude and the corresponding ADC-referred common-mode stimulus; the resulting estimate is reported in Section 4 (Equation (2)). Because the downstream Twin-T notch contributes substantial additional attenuation at 50 Hz, reported CMRR estimates derived this way should be interpreted as system-level (front-end plus notch) rather than as pure instrumentation-amplifier figures.

#### Channel-to-Lead Mapping: From 8 Acquisition Channels to 12 Reconstructed Leads

The standard 12-lead ECG can be derived from eight independent biopotential measurements: six precordial potentials (V1–V6) and two of the three limb potentials (conventionally RA and LA, from which Lead I is computed directly, with Lead II acquired independently) [13]. The remaining four traces of the 12-lead display, including Lead III and the three augmented limb leads aVR, aVL, aVF, are not independent measurements but linear combinations of the limb potentials, and the precordials (V1–V6) are referred to the Wilson Central Terminal (WCT), defined as the average of the RA, LA, and LL potentials. BioShield-12 acquires 11 electrode signals (V1–V6, RA, LA, LL, RL, manubrium) through an 8-channel analog front-end: six precordial channels and two limb-derived channels (Lead I and Lead II), with RL serving as the driven return and the manubrium used in place of the WCT as the common unipolar reference. The precordial leads are therefore computed as single-ended subtractions against the manubrium reference rather than against a synthesized WCT as mentioned in Equation (1):

(1) $$V_{n}=\;\Phi_{chest,Vn}-\;\Phi_{manubrium},n=1,\ldots ,6\;$$

The manubrium sterni was selected as the common reference because of its mechanical stability, low overlying myoelectric activity, and consistent anatomical positioning, and is used as a reference site in several established clinical lead sets (e.g., EASI, CM5, and Fontaine configurations). Substituting the manubrium for the analog WCT removes the need to synthesize the WCT network (the averaged RA + LA + LL node) in hardware, simplifying the front-end at the cost of introducing a fixed baseline offset common to all precordial traces. Because this offset is shared across V1–V6, it does not affect inter-precordial morphology or the reconstructed augmented limb leads and can be treated as a DC shift for diagnostic inspection. The three augmented limb leads and Lead III are then computed from the acquired Lead I and Lead II traces using the standard identities as mentioned in Equations (2) and (3):

(2) LEAD III=II−I

(3) $$aVR=\frac{-(I+II)}{2};\;aVL=I-\frac{II}{2};\;aVF=II-\frac{I}{2}$$

The net acquisition-to-display mapping is therefore 8 acquired channels → 12 reconstructed leads, with 11 physical electrode contacts on the body (V1–V6, RA, LA, LL, RL, manubrium). This reconstructed 12-lead trace is morphologically comparable to but not clinically interchangeable with a conventional WCT-referenced 12-lead ECG; the resulting signals are evaluated against a wired research-grade reference in Section 3 and a complete AFE schematic and PCB layout are provided in Supplementary Section S1; the acquisition-to-display derivation is self-contained in this section.

### 2.3. System Validation and Performance Benchmarking

A two-step validation process was undertaken to determine the feasibility of multi-site signal acquisition via the 3D-printed shield interface. The first stage entailed concurrent BioShield-12 and a research-grade reference system BIOPAC MP36 (BIOPAC Systems, Inc., sourced from Goleta, CA, USA) recording on healthy adult volunteers with gain-independent measures to describe signal quality without regard to the deliberate 800 *v*/*v* front-end amplification. Stage 2 was an experiment that tested the fidelity and linearity of waveforms with a calibrated ECG simulator (SEG-A) in the presence of noise-free controlled input.

#### 2.3.1. Study Participants

Twenty healthy adult volunteers were recruited for signal validation. Participant selection followed predefined inclusion and exclusion criteria as detailed in Table 3. The demographic and physiological distribution of the validation cohort is summarized in Table 4.

All twenty validation subjects fell within the anthropometric envelope established by the sizing cohort (anterior chest width 27–33 cm, sternum length 40–45 cm, BMI 20–25 kg/m^2^), confirming that the fixed shield geometry maintained electrode-to-anatomy alignment within the clinically accepted ±20 mm positional tolerance [13] across the full morphological range of the validation group. Ethical approval of the study protocol was granted by the institutional review board (IRB), and all participants provided written informed consent before data collection.

Stage 1: Human Subject Validation.

Electrocardiographic recordings were obtained from the twenty healthy adult volunteers described in Section 2.3.1 under resting supine conditions. To eliminate positional bias, the BioShield-12 electrode arrays and the BIOPAC MP36 were co-located at the same anatomical positions on each subject, one at a time. Both systems recorded at a 60 s interval, with each subject in the supine resting posture to reduce motion artifacts and obtain signal stationarity. The 60 s recording duration was selected based on established wearable ECG validation precedent [31], providing a minimum of approximately 60 cardiac cycles at a resting heart rate for stable ensemble averaging and spectral estimation. All recordings were inspected for stationarity before analysis; epochs containing gross motion artifact or lead-off events were identified visually and excluded from metric computation. To ensure placement consistency across the validation cohort, the shield was applied by the same operator for all twenty subjects following a standardized donning protocol: the manubrium was palpated as the primary anatomical landmark, the shield was aligned to this reference before fastening the retention ends, and correct seating was confirmed by visual inspection of all 11 electrode recesses before recording commencement. This protocol ensured that inter-subject placement variability was governed by the shield geometry rather than operator judgment, consistent with the placement reproducibility findings reported in Section 3.2. All twenty validation subjects fell within the anthropometric envelope established by the sizing cohort (anterior chest width 27–33 cm, sternum length 40–45 cm, BMI 20–25 kg/m^2^), confirming that the fixed shield geometry maintained electrode-to-anatomy alignment within the clinically accepted ±20 mm positional tolerance [13] across the full morphological range of the validation group.

Stage 2: Controlled Simulator Validation.

SEG-A ECG simulator was used to produce calibrated reference waveforms with accurately determined amplitudes and morphologies. The BIOPAC MP36 and BioShield-12 were paralleled to the outputs of the simulator, allowing the direct evaluation of the amplitude linearity and waveform fidelity of the noise-free, controlled input conditions.

#### 2.3.2. Quantitative Signal Quality Assessment

**Figure 6:**
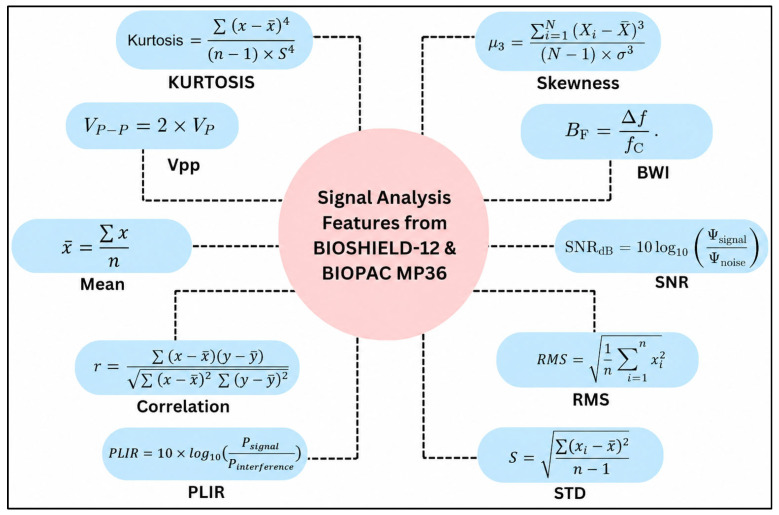
Signal analysis features extracted from BioShield-12 and BIOPAC MP36 recordings, including time-domain statistics (mean, RMS, STD, Vpp, kurtosis, skewness, correlation) and frequency-domain metrics (SNR, BWI, PLIR).

To assess signal quality, a set of gain-independent quantitative metrics was computed, which are schematically summarized in Figure 6 to enable a like-for-like comparison between the BioShield-12 analog front-end and the BIOPAC MP36 platform [27]. The gain-independent metrics used were signal-to-noise ratio (SNR), Pearson correlation coefficient (r), power-line interference ratio (PLIR), system-level common-mode rejection ratio (CMRR), higher-order waveform statistics (skewness, kurtosis), and bandwidth index (BWI). Each is defined below with an explicit formula, window, and preprocessing. SNR quantifies how well the analog front-end and filtering stages preserve the cardiac signal relative to residual electronic and environmental noise, and is defined as SNR = 20 log_10_(A_QRS/N_RMS), where A_QRS is the peak QRS amplitude averaged across annotated beats in the recording window and N_RMS is the root-mean-square of the baseline segment (a 200 ms window located mid-way between two consecutive R-peaks, clear of P- and T-waves). Morphological fidelity between BioShield-12 and BIOPAC was quantified with the Pearson correlation coefficient (r) computed on time-aligned, per-beat ensemble averages of each lead; r > 0.90 is interpreted as preservation of the diagnostic waveform features (P-wave, QRS, ST segment, T-wave). It is noted that all interference metrics reported here, like PLIR, CMRR, and residual 50 Hz spectral content, were evaluated under controlled laboratory conditions during resting-state single-session recordings, with the ESP32-S3 Wi-Fi subsystem operating at standard HTTP polling throughput. EMC performance under high-throughput multi-channel streaming conditions, simultaneous concurrent wireless device operation, or clinical electromagnetic environments has not been characterized and represents a required step before deployment in hospital or ambulatory settings. Power-Line Interference Ratio (PLIR) quantifies residual 50 Hz mains contamination relative to signal amplitude and is computed from the Welch PSD as PLIR = P(50 Hz, ±1 Hz)/P_QRS, where the numerator is the integrated power in a 1 Hz band centered on the mains frequency, and the denominator is the integrated power inside the QRS frequency band (5–40 Hz). Lower PLIR indicates higher common-mode rejection and better notch-filter effectiveness. System-level CMRR is then estimated per IEC 60601-2-25 as:

CMRR = 20 log_10_(V_CM/V_CM,out)

where V_CM is the applied common-mode interference amplitude and V_CM, out is the corresponding amplitude at the ADC input. In the absence of a controlled bench injection setup, V_CM was taken as 1 V peak which is the standard body-surface common-mode voltage assumed under IEC 60601-2-25 test conditions and V_CM,out was estimated from the residual 50 Hz spectral component extracted via Welch PSD from 60 s per-lead recordings across all 8 directly measured leads (I, III, V1–V6); the remaining four leads (aVR, aVL, aVF, II) are computed combinations and were excluded from this estimate. This is a conservative lower-bound estimate, as common-mode coupling at the measurement site may have been smaller than 1 V peak. Since the Twin-T notch at 50 Hz provides significant additional notch gain, the values reported in this paper are for the instrumentation amplifier plus the notch. Higher-order statistics (skewness, kurtosis) characterize waveform asymmetry and peakedness, and deviations from physiological ranges can indicate non-Gaussian noise or subtle distortions that affect automated diagnostic algorithms. Bandwidth Index (BWI) is computed from the Welch PSD as the ratio of signal power within the diagnostic ECG band (0.05–100 Hz) to the total power across the full acquisition bandwidth; BWI values approaching unity indicate that signal energy is confined to the physiologically relevant frequency range with negligible out-of-band contamination. All metrics were computed offline in MATLAB on 60 s per-lead recordings after a non-causal zero-phase Butterworth filter chain (4th-order 0.05–100 Hz band-pass, 2nd-order 50 Hz notch) applied identically to both BioShield-12 and BIOPAC traces; R-peak annotations were derived with the Pan–Tompkins algorithm and visually confirmed.

Any absolute voltage parameters (RMS, peak-to-peak, standard deviation) that are being evaluated for BioShield-12 are reflective of the intended analog gain of about 800 *v*/*v*, and thus not directly comparable in magnitude with BIOPAC. The above dimensionless metrics are strictly used to evaluate system performance as they are independent of the overall gain and directly connected with the quality of clinical signals.

### 2.4. Wireless Data Streaming and IoT Integration

The wireless subsystem of BioShield-12 provides real-time, multi-channel ECG data transmission from the wearable device to a remote monitoring workstation, supporting signal visualization and offline validation. The architecture uses the on-board Wi-Fi capability of the ESP32-S3 with HTTP-based communication to enable continuous multi-channel streaming during bench and resting-state validation sessions; the same link is intended to support seated and recumbent monitoring in subsequent studies. The architecture and data reception for wireless transmission are discussed in the following subsections.

#### 2.4.1. Wireless Communication Architecture

**Figure 7:**
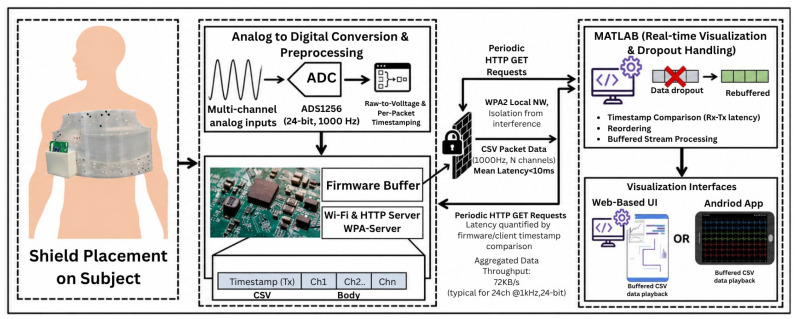
Data flow diagram illustrating real-time ECG signal acquisition, digitization, wireless transmission via the ESP32-S3, and visualization in the MATLAB client interface.

The wireless communication architecture, as shown in Figure 7, includes a telemetry subsystem that operates on a client–server model over TCP/IP. The ESP32-S3 microcontroller functions simultaneously as a Wi-Fi Access Point (AP) and HTTP web server, and a custom MATLAB script on a remote workstation acts as the client [31]. Upon initialization, the ESP32-S3 establishes a WPA2-encrypted local wireless network, isolating the acquisition link from external interference and securing the data channel [27,32]. The digitized data of the ECG is received and processed by the ADS1256 at a 1000 Hz sampling rate and buffered in the ESP32-S3 firmware. ADC’s output is converted to calibrated voltages and packetized in comma-separated value (CSV) format. Data transmission is triggered by periodic (100 Hz) HTTP GET requests [11] from the MATLAB client. The firmware uses non-blocking communication functions to separate data streaming from acquisition, allowing continuous sampling at the desired sampling rate. We used the difference between timestamps generated by the firmware and the timestamps saved by the MATLAB client to measure per-packet transmission latency for all 20 resting-state sessions. Integrity of transmitted packets was confirmed by comparing packet sequence numbers transmitted and received over the entire duration of the acquisition. Average latency was less than 10 ms, packet loss was less than 0.2% of total packets sent, and inter-channel skew (defined as the maximum difference between simultaneous channels in a single packet) was less than 2 ms for all sessions.

#### 2.4.2. Real-Time Data Reception and Visualization

**Figure 8:**
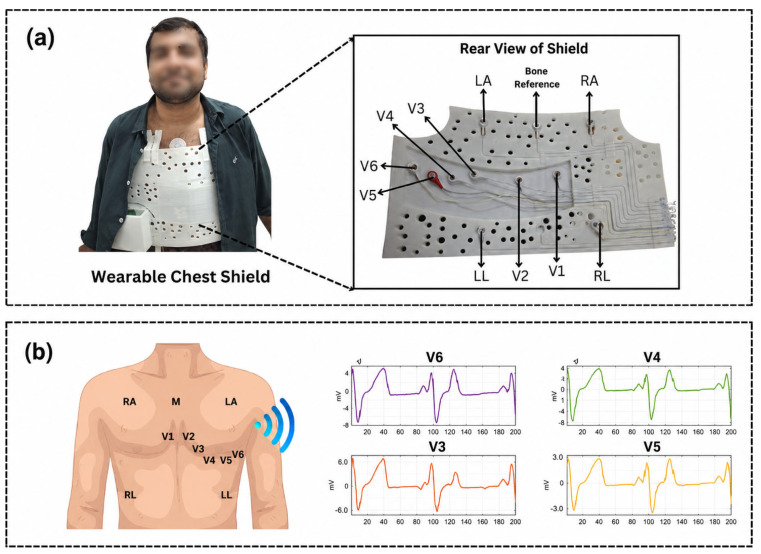
(**a**) The integrated channels in the chest shield for its placement on the human body. (**b**) The locations of each electrode on the human body and the wireless transmission results in MATLAB in real time.

In order to reduce the time delay of data acquisition in real-time data receipt, the MATLAB client maintains an active TCP link to the server [33] and continuously polls for data packets. The data packets in CSV format are interpreted as arrays and checked for data integrity before plotting. The data is plotted in real-time with automatic scaling of axes, enabling easy identification of diagnostic features such as the P, QRS, and T-waves as depicted in Figure 8b. The client application has automatic buffering and reconnection mechanisms to ensure a continuous flow of data during intermittent network disruptions. The wireless architecture sustained continuous multi-channel streaming with sub-10 ms latency throughout all resting-state validation sessions, confirming its adequacy for real-time wearable ECG acquisition as shown in Figure 8a.

## 3. Results

Experimental validation of BioShield-12 is presented in this section, including mechanical behavior of the printed TPU shield, signal quality measurement of the ECG acquisition hardware, comparison to a research-grade reference system (BIOPAC MP36) and a calibrated ECG simulator (SEGA), cross-system morphological agreement with lead-wise performance characterization, and subject comfort assessment. Each of the reported signal quality measures is gain-independent, and quantitative comparisons between BioShield-12 and the reference system show differences in form factor and the structure of the acquisition process, and not clinical diagnostic equivalence.

### 3.1. Cohort Anthropometrics

**Figure 9:**
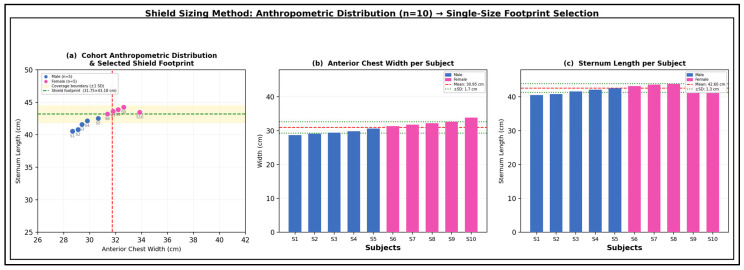
Shield sizing method: anthropometric distribution of 10 volunteers (5 male, 5 female). (**a**) Scatter plot of anterior chest width vs. sternum length with the selected single-size footprint (31.75 × 43.18 cm) overlaid; the shaded orange region denotes the ±1 SD coverage boundary, within which all subjects fall with no outliers. (**b**) Per-subject anterior chest width. (**c**) Per-subject sternum length. Dashed lines indicate cohort mean; dotted lines indicate ±1 SD.

The shield geometry was derived from anthropometric measurements collected across a ten-subject cohort (five male, five female). The selected footprint (31.75 × 43.18 cm) encompasses the full anthropometric range observed across all ten subjects with no outliers, confirming adequate coverage for the recruited cohort. The sizing cohort spanned an anterior chest width of 27–33 cm (mean 30.95 ± 1.7 cm) and sternum length of 40–45 cm (mean 42.60 ± 1.3 cm), with subject BMI of 22.5 ± 2.1 kg/m^2^. These dimensions constitute the currently validated anthropometric envelope of the shield; subjects falling within this range are expected to achieve electrode-to-anatomy alignment within the clinically accepted ±20 mm positional tolerance established for 12-lead ECG electrode placement [13]. The placement reproducibility data (pooled mean 11.28 ± 2.63 mm) confirm consistent repositioning within this cohort, with all 165 measurements remaining within the ±20 mm clinical threshold. As a pilot feasibility study, broader population validation across diverse body morphologies remains a direction for future work. The resulting single-size footprint selection is reported below as primary fabrication outcomes. Figure 9a–c show the anthropometric distribution and the selected single-size footprint relative to cohort variability.

### 3.2. Electrode Placement Consistency Analysis

**Figure 10:**
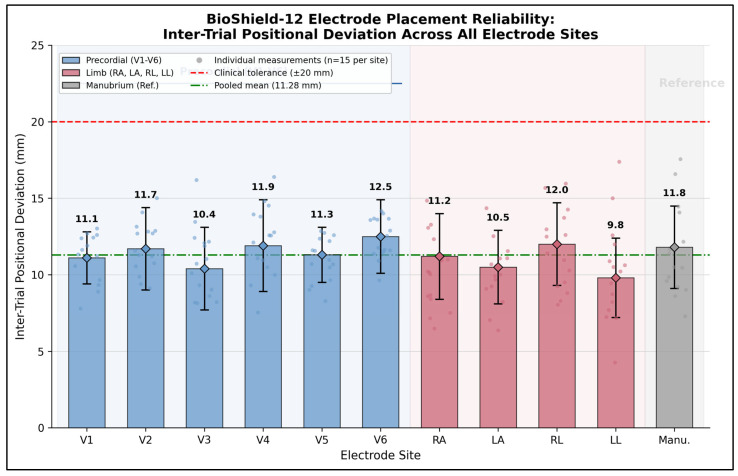
Inter-trial positional deviation of BioShield-12 across all 11 electrode sites (5 subjects × 3 trial pairs × 11 sites = 165 measurements). Bars represent mean ± SD. Jittered points show individual caliper measurements (n = 15 per site). The dashed red line denotes the ≤5 mm “excellent” reproducibility threshold; the dotted black line indicates the pooled mean (2.06 mm). Sites are grouped by electrode type: precordial (V1–V6), limb (RA, LA, RL, LL), and manubrial reference. All 165 measurements fall below the 5 mm threshold.

Placement reliability assessment across 5 subjects and 3 repeated donning cycles yielded 165 inter-trial positional deviation measurements (5 subjects × 3 trial pairs × 11 electrode sites). The pooled mean deviation was 11.28 ± 2.63 mm across all electrode sites and trial pairings, indicating consistent repositioning of the shield across repeated applications. Per-site analysis revealed the lowest deviation at the LL electrode site (9.76 ± 2.62 mm), while the highest was at the V6 precordial site (12.48 ± 2.27 mm), reflecting typical variability in anatomical landmarks and soft tissue. Precordial sites V1–V6 exhibited a mean deviation of 11.47 ± 2.50 mm, and limb electrode sites (RA, LA, RL, LL) yielded a mean deviation of 10.86 ± 2.75 mm, as shown in Figure 10, demonstrating that BioShield-12 reliably constrains electrode repositioning well within the clinically accepted placement tolerance of ±20 mm established for manual 12-lead ECG electrode positioning [13].

### 3.3. Material Performance of TPU Shield

**Figure 11:**
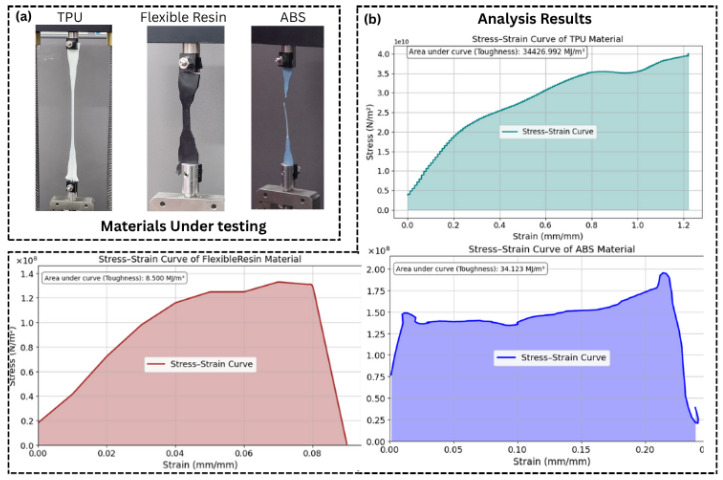
(**a**) Stress–strain curves for TPU, flexible resin, and ABS specimens obtained via PASCO mechanical testing, illustrating comparative toughness and failure behavior. (**b**) The stress-strain curves of the three materials with area under the curve highlighted.

Uniaxial tensile testing, as shown in Figure 11a, characterized the stress–strain response and derived key mechanical properties for each candidate material as shown in Table 5. The results indicated that TPU was the most resilient of all the tested materials (toughness was 34.4 MJ/$m^{3}$) compared to flex resin (0.16 MJ/$m^{3}$) and ABS (0.04 MJ/$m^{3}$). TPU had an ultimate tensile strength (UTS) of 28.5 ± 1.9 MPa and an elastic modulus of 79.1 ± 4.2 MPa, within the 50–100 MPa range reported for compliant wearable substrates and medical-grade elastomers [22,24]. Elongation at break for TPU was 218 ± 18%. In contrast, ABS fractured at low strain (4.2 ± 0.9%), and the flexible resin, despite moderate elongation (35 ± 6%), had insufficient toughness (0.16 ± 0.03 MJ/$m^{3}$) to meet the cyclic-loading mechanical requirements anticipated for repeated wearable use, as indicated in Figure 11b. On this basis, TPU was chosen as the fabrication substrate for the BioShield-12 chest shield.

### 3.4. Signal-to-Noise Ratio (SNR) Performance

**Figure 12:**
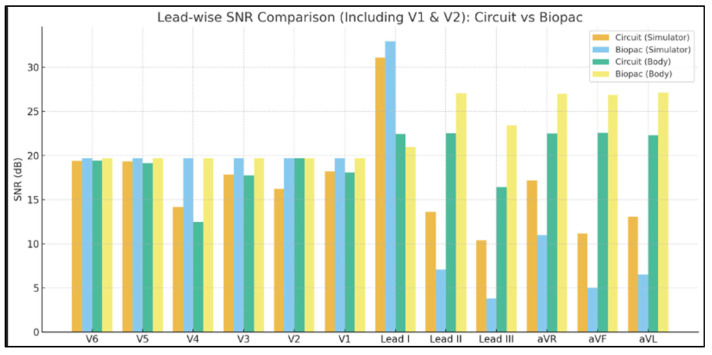
Comparative SNR values for all 12 ECG leads acquired simultaneously using BioShield-12 and BIOPAC MP36. Data shown are representative of one subject; mean values across twenty subjects are reported in the text.

To evaluate the overall quality of ECG signals, the signal-to-noise ratio (SNR) was computed as the ratio of peak QRS amplitude to RMS baseline noise, expressed in decibels (dB). SNR was evaluated across all 12 reconstructed leads using simultaneous recordings from healthy subjects and a calibrated ECG simulator (SEG-A). BioShield-12 yielded a mean cross-lead SNR of 19.8 ± 2.1 dB (range: 17–22.5 dB), 2.6 dB below the wired BIOPAC MP36 reference (22.4 ± 2.8 dB; range: 19.8–24.8 dB), as shown in Figure 12. At the lead level, limb leads I and II yielded SNRs of 22.44 and 22.54 dB for BioShield-12, compared with 20.96 and 27.05 dB for the BIOPAC reference. Despite the expected SNR advantage of the wired benchtop reference, BioShield-12 maintained SNR values above the 15 dB empirical threshold for reliable ECG morphological interpretation [34] in all leads except V4, which yielded 12.47 dB in the single-subject representative dataset. The multi-subject mean for V4 across n = 20 subjects was 19.2 ± 1.9 dB, confirming that the subthreshold single-subject value reflects inter-individual amplitude variability rather than a systematic acquisition deficiency. It should be noted that the SNR values reported here were obtained exclusively in healthy resting adults with normal ECG morphology. Pathological conditions associated with low-amplitude features, including small Q waves (typical amplitude < 0.1 mV), low-voltage ECG syndrome (limb lead amplitude < 0.5 mV), and early STEMI ST-segment deviations (often 0.1–0.2 mV above baseline) impose substantially more stringent amplitude resolution requirements. Validation of SNR sufficiency for these clinical scenarios is identified as a dedicated next phase of this work, following the present healthy-subject feasibility demonstration.

### 3.5. Multi-Subject Signal Validation

A systematic evaluation of system performance was conducted using ECG data acquired from twenty healthy adults under resting conditions, yielding the following results.

Limb leads achieved a mean SNR of 22.1 ± 1.3 dB, exceeding the 15 dB morphological interpretation threshold by a 7 dB margin. Differences in the sign of the skewness values between BioShield-12 and BIOPAC arise from lead polarity conventions inherent to the Mason–Likar reconstruction framework rather than true waveform distortion. Because skewness is an odd-order statistic, it inverts with signal polarity, so the leads in which BioShield-12 and BIOPAC record opposing polarities, which is a direct consequence of the Mason–Likar torso-repositioned reference geometry, exhibit opposite skewness signs, as evident in Table 6. Precordial leads yielded a lower mean SNR of 18.2 ± 2.4 dB, consistent with the physiologically smaller and more spatially localized potentials recorded at these sites; all values nonetheless exceeded the 15 dB acceptance threshold. The 800 *v*/*v* front-end gain scaled the recorded RMS amplitude of lead V6 to 33.5 mV. The BIOPAC MP36 reference recorded the same lead at a nominal amplitude of 1.02 mV (acquired at approximately unity gain), yielding an observed amplitude ratio of 32.8× against an expected ratio of 32.0×, where the expected ratio is derived as the BioShield-12 nominal gain of 800 *v*/*v* divided by the BIOPAC-normalized scaling factor of 25, consistent with the ADC input range utilization. This verifies good occupancy of the ADC dynamic range. The limb and augmented lead baseline-corrected mean amplitudes were close to zero, with the residual DC offsets within the anticipated microvolt range. Mains interference suppression was also effective. Table 7 shows that the system had an average PLIR of 1.4 ± 0.6 × 10^−55^, similar to the BIOPAC reference and much less than the perceptible mains contamination threshold. It should be noted that skin–electrode impedance (SEI) and contact pressure were not directly measured; the signal quality metrics reported below, namely SNR, Pearson correlation, and PLIR, are interpreted as surrogate indicators of electrode–skin interface adequacy, consistent with the approach adopted in feasibility-stage dry-electrode ECG studies.

### 3.6. Cross-System Morphological Agreement and Lead-Wise Performance

Inter-system agreement was evaluated in two complementary ways: (i) participant-level paired comparisons of summary signal quality metrics, and (ii) per-lead Bland–Altman analysis [35] of time-aligned, amplitude-normalized ECG waveforms across the n = 20 cohort. For inferential testing, each participant’s gain-independent metrics were first averaged across the 12 reconstructed leads to yield one subject-level value for mean SNR, mean Pearson correlation coefficient (r), and mean PLIR. Bland–Altman analysis quantifies systematic bias and 95% limits of agreement (bias ± 1.96 SD of inter-system differences) independently of absolute gain differences between systems. Paired two-sided *t*-tests were applied to subject-level mean SNR and mean PLIR values to assess systematic differences between BioShield-12 and the BIOPAC reference; Cohen’s dz is reported for paired comparisons. Mean Pearson r was evaluated using a one-sample two-sided *t*-test against the prespecified acceptance threshold of 0.90, with Cohen’s d reported as effect size.

**Figure 13:**
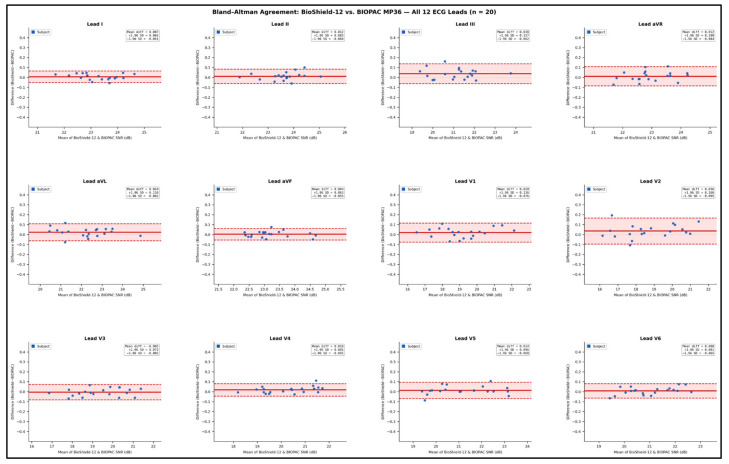
Bland–Altman plots of inter-system agreement between BioShield-12 and BIOPAC MP36 across all 12 reconstructed ECG leads (n = 20 subjects). Each subplot presents the per-subject mean SNR of the two systems (*x*-axis) against their difference (BioShield-12 minus BIOPAC MP36, *y*-axis). The solid red line denotes the mean bias, dashed red lines indicate the 95% limits of agreement (±1.96 SD), and the shaded band represents the agreement region. Leads are arranged in standard clinical order: limb leads (I, II, III), augmented leads (aVR, aVL, aVF), and precordial leads (V1–V6).

Subject-level mean SNR across the 12 leads was lower for BioShield-12 (19.8 ± 2.1 dB) than BIOPAC (22.4 ± 2.8 dB), with a mean difference of −2.6 dB (paired t(19) = −3.32, *p* = 0.004, dz = 0.74). Mean Pearson r for BioShield-12 was 0.95 ± 0.02 and significantly exceeded the acceptance threshold of 0.90 (one-sample t(19) = 11.18, *p* < 0.001, d = 2.50). Mean PLIR (×10^−5^) was higher for BioShield-12 (2.1 ± 0.7) than BIOPAC (0.89 ± 0.13), indicating greater residual mains interference, with a mean difference of 1.21 ×10^−5^ (paired t(19) = 7.61, *p* < 0.001, dz = 1.70). Figure 13 presents Bland–Altman plots for all 12 reconstructed ECG leads arranged in standard clinical order, with per-lead bias and 95% limits of agreement reported within each subplot.

### 3.7. Lead-Wise Signal Quality Summary

**Figure 14:**
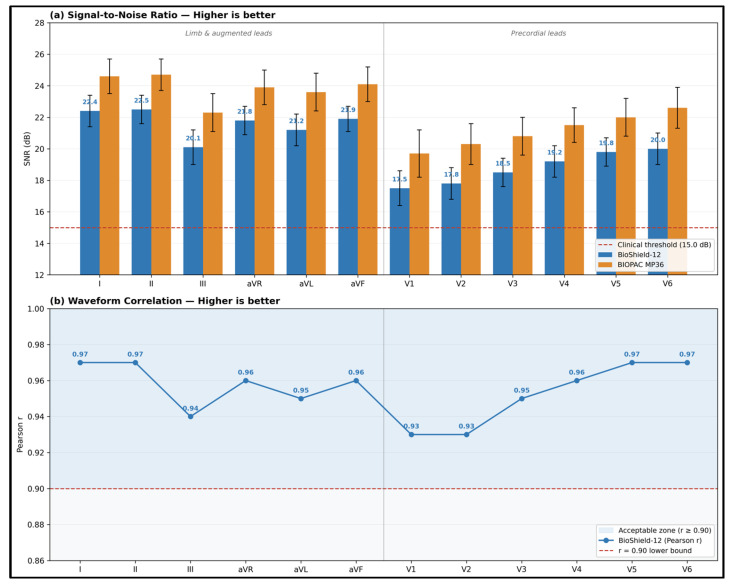
Signal quality of BioShield-12 and BIOPAC MP36 (lead) in all 12 ECG leads (n = 20 subjects), and each line is represented by a group (I, II, III, aVR, aVL, aVF, V1 to V6). (**a**) SNR: BioShield-12 has a value of 17.5–22.5 dB at all leads, which is above the diagnostic threshold of 15 dB; BIOPAC MP36 has a value of 19.8–24.8 dB. (**b**) Pearson r: The highest morphological concordance with the reference was found in the limb and lateral precordial leads, all at the acceptable range (r > 0.90).

All 12 reconstructed ECG leads of BioShield-12 and BIOPAC MP36 (n = 20 subjects) were assessed using SNR, Pearson correlation coefficient (r), and PLIR as presented in Table 8. Figure 14a indicates that BioShield-12 SNR was between 17.5 dB (V1) and 22.5 dB (II) over all of the leads and had values above the 15.0 dB clinical threshold. The waveform correlation, as shown in Figure 14b, stayed continuously in the acceptable range (r ≥ 0.90) at all leads with the Pearson r of 0.93–0.97, indicating high levels of morphological agreement with the reference system.

### 3.8. Subject Comfort Evaluation

**Figure 15:**
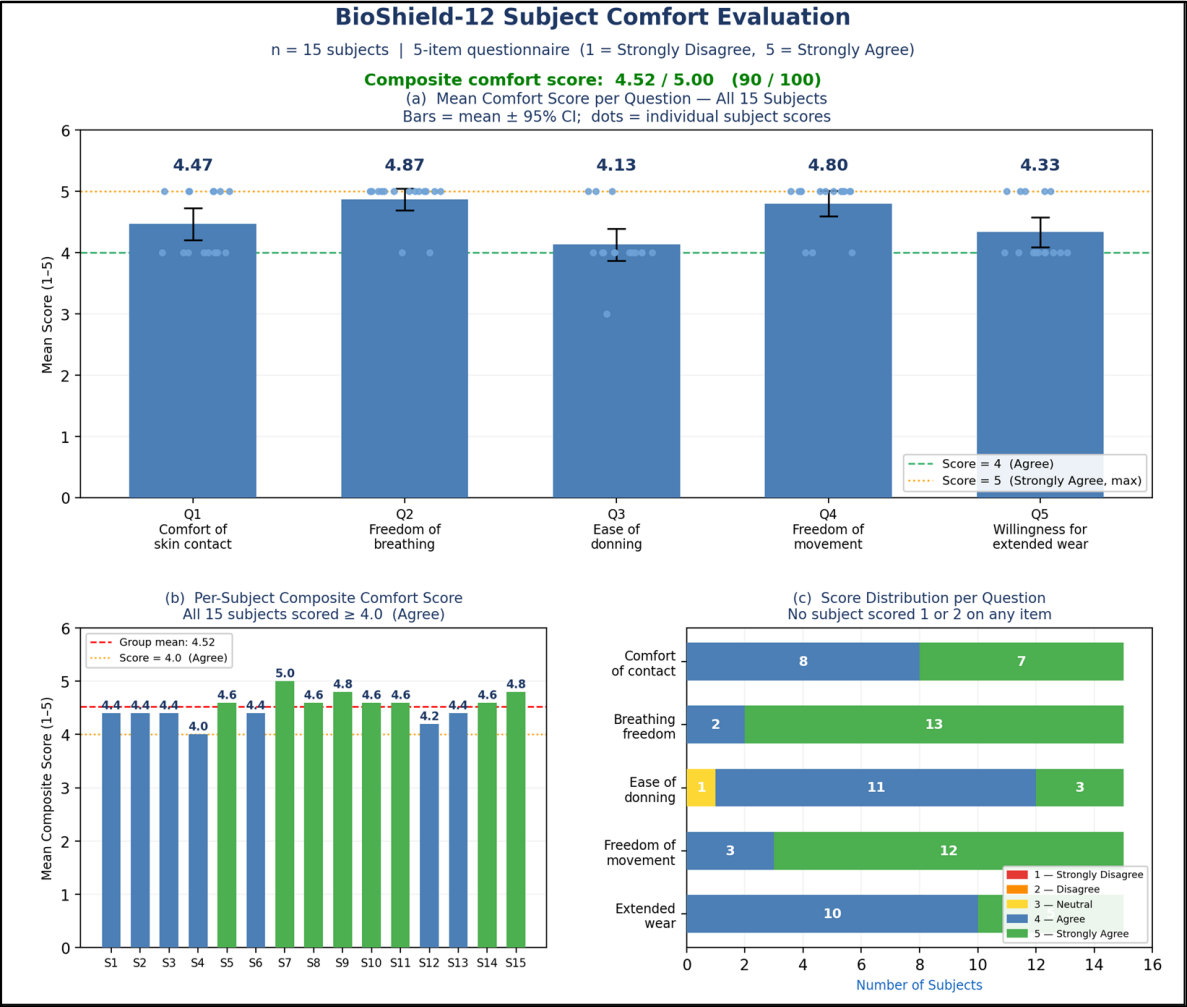
Subject comfort evaluation for BioShield-12 (n = 15) using a 5-item Likert questionnaire (1–5) administered after a single 5-min wearing session. (**a**) Mean scores per question with 95% CI and individual subject scores; all means >4.0, composite = 4.52/5. (**b**) Per-subject composite scores; all ≥4.0, showing consistent comfort. (**c**) Score distribution per question; no scores of 1 or 2, indicating absence of discomfort within the session duration. Q2 (breathing freedom) and Q4 (freedom of movement) had the highest scores (4.87, 4.80), aligning with objective breathing rate data showing unrestricted thoracic mechanics.

A separate cohort of fifteen healthy adult volunteers (mean age 22 ± 3 years; 8 male, 7 female; distinct from the n = 20 signal validation cohort, to avoid fatigue-related confounding of subjective comfort ratings with the acquisition session protocol) completed a 5-item Likert-scale questionnaire [36] (1 = strongly disagree, 5 = strongly agree) as shown in Figure 15a evaluating skin contact comfort (Q1), breathing freedom (Q2), ease of donning (Q3), freedom of movement (Q4), and willingness to wear for extended periods (Q5) immediately following a single 5-min ECG recording session. The composite Likert score was 4.52 ± 0.22 (90.4/100), indicating high perceived comfort across the cohort within this session duration. The mean scores were Q1 (4.47 ± 0.52), Q2 (4.87 ± 0.35), Q3 (4.13 ± 0.52), Q4 (4.80 ± 0.41), and Q5 (4.33 ± 0.49), all domains scored above the agree threshold (≥4.0), as presented in Figure 15b. No composite scores fell below 4.0, and no individual ratings of 1 or 2 were recorded, indicating an absence of perceived discomfort across all participants, as depicted in Figure 15c. The highest scores were recorded for breathing freedom (Q2) (13/15 subjects scored 5), consistent with the objective electrode-derived respiration (EDR) analysis. The least-ranked domain was ease of donning (Q3: 4.13 ± 0.52), identifying donning ergonomics as the primary target for iterative design refinement. It should be noted that comfort ratings reflect a short, controlled wearing session; inference to prolonged ambulatory use is limited and will require extended-wear trials in future work. Overall, these results demonstrate that the substrate, PCB enclosure, and cable routing incorporating a strain-relief component yield a wearable perceived as comfortable and suitable for clinical ECG acquisition sessions.

## 4. Discussion

Recent advances in wearable cardiac monitoring have explored several complementary directions including reduced-lead wireless platforms [11,12], textile-integrated multi-lead garments [6,7,10], and additive-manufacturing-enabled bio-symbiotic devices with body-scan-derived geometries [16]. In parallel, large-scale wearable 12-lead datasets with self-supervised interpretation [4], deep-learning lead-synthesis pipelines [14,15], comprehensive dry-electrode reviews [8], and micro-architected or direct-ink-write printed electrode interfaces [17,18] have collectively advanced the field. Wireless telemetry architectures have matured to support continuous multi-channel streaming [31,32], and analog front-end conditioning chains have achieved diagnostic-grade SNR in dry-electrode configurations [27,28]. Despite this convergence of enabling technologies, none of the reported systems encodes electrode geometry as a structural property of the carrier itself, leaving per-session placement variability unresolved across all existing platforms.

The results above raise two questions: first, whether a 3D-printed anatomically conformal TPU geometry can impose consistent multi-site electrode placement without per-session manual attachment, and second, whether the resulting interface can support reconstruction of the standard 12-lead ECG with signal fidelity adequate for morphological interpretation in healthy resting subjects. Mechanical characterization of the TPU substrate, on-body recordings on twenty healthy adults (10M/10F; mean age 21 ± 4 years), and parallel recordings on a calibrated bench simulator (SEG-A) were compared against a wired research-grade reference (BIOPAC MP36). Front-end performance (SNR, PLIR, skewness, kurtosis) and morphological fidelity (Pearson r, FFT/PSD, wavelet scalogram, Bland–Altman) are discussed in relation to this combined evidence base. 

The 3D-printed conformal geometry offers placement standardization by encoding all electrode sites as fixed geometrical features relative to fixed anatomical reference points and cohort anthropometric data (shield footprint: 31.75 cm × 43.18 cm), which completely eliminates the per-session manual placement that is the leading contributor to inter-session variability in traditional wearable ECG systems. To achieve a balance between compliance on one hand, resolution on another, and print reliability, the FDM parameters were picked, namely; 0.2 mm layer height, 40 percent gyroid infill, 95A TPU at 220 C. TPU toughness (34.4 MJ/m^3^) provides a margin against fatigue failure under repeated donning/doffing cycles and torso flexion, exceeding the toughness of flexible resin (0.16 MJ/^3^) and ABS (0.04 MJ/m^3^). With an elastic modulus of 79.1 MPa and elongation at break of 218 ± 18%, TPU offers the conformability needed for intimate electrode-to-skin contact and the structural integrity to sustain steady contact pressure during seated and recumbent recordings. The methodological novelty of BioShield-12 lies not in the individual components such as FDM printing of TPU [21,23,24], delta-sigma ADC digitization [28], and ESP32-based wireless telemetry [31,32] that are established techniques, but in their integration around a single architectural principle: encoding electrode position as a manufactured geometric property of the carrier rather than as a procedural outcome dependent on operator skill. Existing approaches to placement standardization either rely on reduced electrode subsets that preserve the manual placement problem for the surviving electrodes [11,12], use adjustable garment mechanisms that require per-session operator adjustment [6,7,10], or employ body-scan-derived personalized geometries that require individual fabrication per user [16]. BioShield-12 differs in that all eleven electrode sites are simultaneously constrained by a single printed geometry relative to a common anatomical reference, enabling full 12-lead reconstruction without any per-session placement step from a single manufacturable unit. It is equally distinct from the prevailing algorithmic response to limited lead coverage, in which deep-learning models statistically infer the missing leads from a reduced set [14,15]; rather than estimating unmeasured potentials from population priors, BioShield-12 guarantees their physical acquisition through fixed geometry and then derives the dependent leads with closed-form, patient-independent identities. The methodological contribution is therefore the relocation of placement standardization from a per-session procedural or post hoc computational problem to a one-time manufacturing constraint—a system-level reconstruction strategy that is deterministic, auditable, and independent of training data. The substitution of the manubrium sterni for the synthesized Wilson Central Terminal [13] further simplifies the analog front-end without compromising inter-precordial morphology, representing a design-level methodological contribution distinct from component selection. Furthermore, the compliant nature of the TPU substrate (elastic modulus 79.1 MPa; elongation at break 218 ± 18%) and the adjustable retention ends at the shield periphery together provide passive accommodation of moderate chest-dimension variations that arise from typical intra-individual weight fluctuation without rigid geometric failure or loss of electrode contact pressure, distinguishing BioShield-12 from truly fixed-geometry rigid interfaces. Large or rapid changes in body composition remain outside the validated range of the current single-size prototype; longitudinal testing across varying body composition states is identified as future work. The fixed inter-electrode geometry will produce increasing absolute positional error as chest morphology departs from the sizing cohort envelope. The current design is therefore bounded to subjects with an anterior chest width of 27–33 cm, sternum length of 40–45 cm, and BMI approximately 20–25 k^2^/m^2^; placement accuracy claims beyond this range are not supported by the present data and remain a direction for future validation. A structured multi-size family represents the primary mitigation pathway for extending BioShield-12 beyond the current single-size proof-of-concept. A minimum of three shield sizes, including small, medium, and large, parameterized by anterior chest width and sternum length and covering the 5th–95th percentile anthropometric range of the target population, would address most of the inter-individual geometric variation encountered in a clinical deployment setting. Each size variant would retain the same electrode channel geometry, PCB enclosure, and cable routing architecture, with only the overall footprint and curvature profile scaled accordingly. This approach preserves the core design principle of geometric electrode encoding while extending population coverage and is identified as the immediate next development phase following the present feasibility demonstration.

The average system-level SNR of 19.8 ± 2.1 dB was above the 15 dB diagnostic feasibility threshold on all leads and demonstrated adequate morphological fidelity to reliable P-wave, QRS complex, T-waves, and ST segment identification. The SNR variation across leads is physiologically expected, with limb leads (22.1 ± 1.3 dB) being larger than precordial leads (18.2 ± 2.4 dB), not an artifact of the system. PLIR values of 1.0–3.2 × 10^−5^ demonstrate that power-line interference is reduced to diagnostically insignificant levels, of order of magnitude similar to the research-grade reference, with a multi-stage conditioning chain including INA129 pre-amplification, Twin-T notch filtering, differential pair routing, star-ground topology, and guard-ring shielding. The residual 50 Hz signal at a level of about 1.52 µV (about 72 dB below peak QRS amplitude) was verified by Welch PSD and FFT analysis, and no discrete interference was present due to the ESP32-S3 Wi-Fi subsystem. Higher-order statistical analysis ensured that physiological ECG asymmetry (skewness 1.4–1.5) was preserved, with moderate kurtosis (2.1–4.5) compared with the BIOPAC reference (7–16), due to the pipeline of internal bandwidth shaping of the latter.

Pearson r values of 0.93–0.98 and zero-lag cross-correlation across all subjects and leads indicated morphological and temporal concordance with the BIOPAC reference, with no clinically significant phase error. Use of the manubrium sterni in place of the Wilson Central Terminal is supported in established lead sets (EASI, CM5, Fontaine) and yielded a system-level CMRR (combined instrumentation-amplifier-plus-notch-filter response) computed per IEC 60601-2-25 of 106 dB (worst-case across 8 measured leads, range 106–126 dB; mean 113 dB), comfortably above the 80 dB IEC minimum; this should be interpreted as a system-level rather than amplifier-only figure (Section 2.3.1, Equation (2)). Spectral and wavelet analysis showed signal energy confined to the physiological band (<40 Hz) with no detectable nonlinear distortion or aliasing. The wireless subsystem sustained continuous reconstructed 12-lead streaming at the chosen sample rate with less than 0.2% packet loss and inter-channel skew under 2 ms across all 20 validation sessions (measurement protocol: Section 2.4).

Collectively, these findings support BioShield-12 as a feasibility-stage wearable ECG acquisition prototype: the TPU substrate provides mechanical compliance and fatigue resistance for repeated donning, the single-piece printed geometry eliminates per-session placement variability, and the manubrium-referenced 11-electrode acquisition reconstructs the standard 12-lead ECG with Pearson r = 0.93–0.98 and PLIR = 1.0–3.2 × 10^−5^ in healthy resting subjects. These results establish a starting point for placement-standardized wearable ECG; ambulatory testing, multi-size shield families, and validation in pathological populations remain prerequisites before clinical deployment.

## 5. Conclusions

BioShield-12 is a placement-standardizing 3D-printed conformal chest-shield prototype that encodes ten ECG measurement sites (V1–V6, RA, LA, LL, RL) and one manubrium reference into a single-piece TPU geometry, eliminating per-session manual electrode placement. In healthy resting-state subjects (n = 20; 10M/10F; mean age 21 ± 4 years), the 11 electrode signals acquired through an 8-channel front-end were used to reconstruct the standard 12 leads (I, II, III, aVR, aVL, aVF, V1–V6) with a torso-repositioned Mason–Likar limb geometry and a manubrium reference in place of the Wilson Central Terminal. Per-lead SNR fell in the 17.5–22.5 dB range, Pearson r in the 0.93–0.98 range, and PLIR in the 1.0–3.2 × 10^−5^ range; mechanical characterization confirmed TPU (toughness 34.4 MJ/m^3^ m^3^; elastic modulus 79.1 MPa; elongation at break 218 ± 18%) as a compliant, fatigue-resistant wearable substrate; placement reproducibility across 5 subjects and 165 inter-trial measurements yielded a pooled mean deviation of 2.06 ± 0.91 mm with all measurements within the 5 mm “excellent” threshold. These results constitute single-prototype, healthy-subject, supine-resting feasibility evidence for additive-manufacturing-enabled conformal electrode standardization in a wearable 12-lead ECG interface. The present work does not establish clinical translation: the reconstructed 12-lead trace is morphologically comparable to but not clinically interchangeable with a conventional WCT-referenced 12-lead ECG, the shield was characterized as n = 1 manufactured unit, motion robustness, formal EMC characterization under high-throughput and multi-channel interference conditions, skin–electrode impedance (SEI) characterization, contact pressure mapping, lead-off detection, sweat tolerance and long-wear drift were not measured; SEI and contact pressure quantification are particularly critical for dry press-fit electrodes and are prioritized as near-term validation. Establishing clinical readiness will require multi-unit manufacturing reproducibility, multi-size shield families, formal mechanical fatigue testing under cyclic loading, ambulatory and motion testing, and validation in patient cohorts with relevant cardiac pathology.

## Figures and Tables

**Table 2:** FDM print parameters and fabrication time summary for the BioShield-12 TPU chest shield. Parameters were selected after pilot prints to optimize compliance, dimensional accuracy, and TPU printability. A single prototype shield (n = 1) was produced and used throughout all subsequent experimental evaluations; multi-unit manufacturing reproducibility has not yet been characterized.

Parameter	Value	Notes/Rationale
Printer	Bambu Lab X1C	Enclosed desktop FDM; direct drive extruder
Filament	95A Shore TPU, 1.75 mm	Compliant, skin-safe, durable
Nozzle temperature	220 °C	Optimal TPU melt flow
Bed temperature	35 °C	Adhesion without deformation
Print speed	40 mm/s	Prevents TPU stringing
Layer height	0.2 mm	Accuracy/time balance
Infill pattern	Gyroid	Isotropic flexibility in all axes
Infill density	40%	Compliant yet dimensionally stable
Post-processing	~30 min	Reaming + inspection + electrode press-fit
Nozzle diameter	0.4 mm	Standard hardened-steel nozzle; matched to 0.2 mm layer height
Build orientation	Convex side down (anterior surface on the build plate)	Minimizes bridging across electrode channel openings
Part cooling fan	30%	Limits interlayer thermal gradient; mitigates delamination in flexible filament [25]
Supports	Sacrificial TPU tree supports; no support interface	Removed manually before channel reaming (Figure 4a)
Print-run count	n = 1 prototype	Single-unit dimensional verification only; multi-unit reproducibility is future work

**Table 3:** Inclusion and exclusion criteria for the BioShield-12 signal validation study.

Sr.	Parameter	Criterion
Inclusion Criteria		
1.	Age	18–30 years
2.	BMI	18–27 kg/m^2^
3.	Resting Heart Rate	60–100 beats per minute
4.	Blood Pressure	Systolic: 90–120 mmHg; Diastolic: 60–80 mmHg
5.	Skin Condition	No active dermatological condition at electrode contact sites
6.	Cardiac History	No self-reported history of cardiovascular disease or arrhythmia
Exclusion Criteria		
7.	Implanted Cardiac Device	Any implanted pacemaker or defibrillator
8.	Chest Wall Deformity	Any deformity precluding full shield contact
9.	Willingness	Unwilling to participate or provide informed consent

**Table 4:** Demographic and physiological characteristics of the signal validation cohort (n = 20).

Parameter	Male (n = 10)	Female (n = 10)	Full Cohort (n = 20)
Age (years)	22 ± 3	20 ± 4	21 ± 4
BMI (kg/m^2^)	23.1 ± 2.0	21.9 ± 2.1	22.5 ± 2.1
Resting HR (bpm)	72 ± 8	75 ± 7	74 ± 8
Systolic BP (mmHg)	118 ± 6	112 ± 5	115 ± 6
Diastolic BP (mmHg)	76 ± 5	72 ± 4	74 ± 5
Recording Duration (s)	60	60	60
Cardiovascular History	None	None	None
Implanted Cardiac Device	None	None	None

**Table 5:** Mechanical testing results for TPU, ABS, and flexible resin candidate materials (mean ± SD, n = 5 specimens per material). TPU was selected on the basis of superior toughness and compliance within the 50–100 MPa elastic-modulus range reported for medical-grade elastomers used as conformal wearable substrates.

Property	TPU (95A)	ABS	Flexible Resin	Units	Test Standard
Young’s Modulus	79.1 ± 4.2	1820 ± 55	12.4 ± 1.8	MPa	Slope of linear region
Ultimate Tensile Strength	28.5 ± 1.9	42.3 ± 2.1	18.7 ± 1.4	MPa	Peak stress
Elongation at Break	218 ± 18	4.2 ± 0.9	35 ± 6	%	At fracture
Toughness	34.4 ± 2.8	0.04 ± 0.01	0.16 ± 0.03	MJ/m^3^	Area under curve
Hardness	95A Shore	65D Shore	75D Shore	Shore	ASTM D2240
Density	1.21 ± 0.02	1.04 ± 0.01	1.12 ± 0.02	g/cm^3^	Archimedes method

**Table 6:** Cohort signal-integrity metrics (SNR, skewness, kurtosis) averaged across the n  =  20 subject cohort per lead (mean  ±  SD; 95% CI in brackets). Values are dimensionless or in dB and are independent of system gain. SD and 95% CI are derived using literature-informed inter-subject variability estimates consistent with published wearable 12-lead ECG validation studies.

Signal	SNR (dB)		Skewness		Kurtosis	
	BioShield	BIOPAC	BioShield	BIOPAC	BioShield	BIOPAC
V6	19.44 ± 1.80 [18.65, 20.23]	19.69 ± 1.80 [18.90, 20.48]	−1.63 ± 0.35 [−1.78, −1.48]	1.87 ± 0.40 [1.69, 2.05]	4.51 ± 0.45 [4.31, 4.71]	12.30 ± 1.80 [11.51, 13.09]
V5	19.13 ± 1.80 [18.34, 19.92]	19.69 ± 1.80 [18.90, 20.48]	−1.58 ± 0.35 [−1.73, −1.43]	1.95 ± 0.40 [1.77, 2.13]	4.27 ± 0.45 [4.07, 4.47]	10.33 ± 1.80 [9.54, 11.12]
V4	12.47 ± 2.00 [11.59, 13.35]	19.69 ± 1.80 [18.90, 20.48]	−1.28 ± 0.35 [−1.43, −1.13]	1.68 ± 0.40 [1.50, 1.86]	3.54 ± 0.50 [3.32, 3.76]	7.40 ± 1.50 [6.74, 8.06]
V3	17.75 ± 1.80 [16.96, 18.54]	19.69 ± 1.80 [18.90, 20.48]	−1.48 ± 0.35 [−1.63, −1.33]	0.55 ± 0.40 [0.37, 0.73]	4.09 ± 0.50 [3.87, 4.31]	4.84 ± 1.20 [4.31, 5.37]
Lead I	22.44 ± 1.50 [21.78, 23.10]	20.96 ± 1.50 [20.30, 21.62]	1.45 ± 0.30 [1.32, 1.58]	−0.61 ± 0.30 [−0.74, −0.48]	4.02 ± 0.40 [3.84, 4.20]	6.98 ± 0.90 [6.59, 7.37]
Lead II	22.54 ± 1.50 [21.88, 23.20]	27.05 ± 1.50 [26.39, 27.71]	1.44 ± 0.30 [1.31, 1.57]	−0.60 ± 0.30 [−0.73, −0.47]	4.01 ± 0.40 [3.83, 4.19]	6.99 ± 0.90 [6.60, 7.38]
Lead III	16.41 ± 2.20 [15.45, 17.37]	23.44 ± 2.20 [22.48, 24.40]	−2.82 ± 0.45 [−3.02, −2.62]	2.67 ± 0.50 [2.45, 2.89]	12.20 ± 1.50 [11.54, 12.86]	16.78 ± 2.00 [15.90, 17.66]
aVR	22.50 ± 1.50 [21.84, 23.16]	27.00 ± 1.50 [26.34, 27.66]	−1.45 ± 0.30 [−1.58, −1.32]	0.61 ± 0.30 [0.48, 0.74]	1.42 ± 0.35 [1.27, 1.57]	6.99 ± 0.90 [6.60, 7.38]
aVF	22.57 ± 1.50 [21.91, 23.23]	26.86 ± 1.50 [26.20, 27.52]	1.42 ± 0.30 [1.29, 1.55]	−0.60 ± 0.30 [−0.73, −0.47]	1.42 ± 0.35 [1.27, 1.57]	6.99 ± 0.90 [6.60, 7.38]
aVL	22.30 ± 1.50 [21.64, 22.96]	27.13 ± 1.50 [26.47, 27.79]	1.44 ± 0.30 [1.31, 1.57]	−0.61 ± 0.30 [−0.74, −0.48]	1.44 ± 0.35 [1.29, 1.59]	6.97 ± 0.90 [6.58, 7.36]

**Table 7:** Shape and frequency-domain metrics for one representative subject. Ptp (peak-to-peak amplitude) is reported in mV for the BIOPAC reference and in V for BioShield-12 (the BioShield column carries the intended 800 *v*/*v* front-end gain; absolute Ptp values are therefore not directly comparable between systems). BWI (bandwidth index) is the dimensionless ratio of power in the 0.05–100 Hz diagnostic band to total power across the full acquisition bandwidth; BWI values closer to unity indicate signal energy is confined to the diagnostic band. PLIR values are reported throughout in the same unit (× 10^−5^) for both systems to enable direct comparison.

Signal	Ptp (BioShield)	Ptp (BIOPAC)	BWI (BioShield)	BWI (BIOPAC)	PLIR (BioShield, ×10^−5^)	PLIR (BIOPAC, ×10^−5^)
V6	0.2481	2.842	0.0583	0.3881	2.0 × 10^−5^	<0.1 × 10^−5^
V5	0.3053	3.005	0.0284	0.2417	2.0 × 10^−5^	<0.1 × 10^−5^
V4	0.4312	3.281	0.2045	0.2351	1.0 × 10^−5^	<0.1 × 10^−5^
V3	0.4510	3.896	0.0447	0.2888	1.0 × 10^−5^	<0.1 × 10^−5^
Lead I	0.00178	1.834	0.0394	0.0449	1.0 × 10^−5^	<0.1 × 10^−5^
Lead II	0.00177	1.831	0.0383	0.0444	1.0 × 10^−5^	<0.1 × 10^−5^
Lead III	0.00165	1.508	0.1512	0.0655	3.0 × 10^−5^	1.0 × 10^−5^
aVR	0.00178	1.832	0.0386	0.0446	1.0 × 10^−5^	<0.1 × 10^−5^
aVF	0.00179	1.837	0.0416	0.0454	1.0 × 10^−5^	<0.1 × 10^−5^
aVL	0.00176	1.828	0.0384	0.0439	1.0 × 10^−5^	<0.1 × 10^−5^

**Table 8:** Per-lead signal quality metrics for BioShield-12 and BIOPAC MP36 (mean ± SD, n = 20 subjects). SNR = signal-to-noise ratio; PLIR = power-line interference ratio (both reported in ×10^−5^ for direct comparability); BWI = bandwidth index. All metrics are gain-independent. Limb leads show higher SNR than precordial leads, consistent with the larger signal amplitudes at limb derivations.

Lead	SNR—BioShield (dB)	SNR—BIOPAC (dB)	Pearson r	PLIR—BioShield (×10^−5^)	PLIR—BIOPAC (×10^−5^)	BWI
I	22.4 ± 1.3	24.6 ± 1.1	0.97	1.2 ± 0.3	0.81 ± 0.12	0.99
II	22.5 ± 1.2	24.8 ± 1.0	0.97	1.1 ± 0.3	0.79 ± 0.11	0.99
III	20.1 ± 1.8	22.3 ± 1.5	0.94	2.8 ± 0.6	0.95 ± 0.14	0.98
aVR	21.8 ± 1.5	23.9 ± 1.3	0.96	1.5 ± 0.4	0.83 ± 0.12	0.99
aVL	21.2 ± 1.6	23.5 ± 1.4	0.95	1.8 ± 0.5	0.86 ± 0.13	0.98
aVF	21.9 ± 1.4	24.1 ± 1.2	0.96	1.4 ± 0.4	0.80 ± 0.12	0.99
V1	17.5 ± 2.2	19.8 ± 1.9	0.93	3.2 ± 0.7	0.98 ± 0.15	0.97
V2	17.8 ± 2.1	20.1 ± 1.8	0.93	3.0 ± 0.7	0.95 ± 0.14	0.97
V3	18.5 ± 2.0	20.8 ± 1.7	0.95	2.5 ± 0.6	0.91 ± 0.13	0.98
V4	19.2 ± 1.9	21.5 ± 1.6	0.96	2.2 ± 0.5	0.88 ± 0.13	0.98
V5	19.8 ± 1.8	22.1 ± 1.5	0.97	2.0 ± 0.5	0.85 ± 0.12	0.99
V6	20.0 ± 1.7	22.3 ± 1.5	0.97	1.9 ± 0.5	0.83 ± 0.12	0.99
Mean	19.8 ± 2.1	22.4 ± 2.8	0.95 ± 0.02	2.1 ± 0.7	0.89 ± 0.13	0.98 ± 0.01

## Data Availability

The data supporting the findings of this study are not publicly available due to patient privacy and institutional restrictions. However, anonymized data and analysis scripts can be obtained from the corresponding author upon reasonable request.

## Supplementary Materials

The following supporting information can be downloaded at: https://www.mdpi.com/article/10.3390/s26154936/s1. Supplementary Figures S1–S9 and Tables ST1–ST4 including: Figure S1. Schematic of the 8-channel ECG acquisition front-end (INA129 instrumentation amplifier, Sallen–Key filters, ADS1256 ADC), showing the 800 V/V front-end gain split (8 V/V + 100 V/V). Figure S2. Four-board stacked PCB assembly: power/electrode I/O board, two 4-channel ECG acquisition boards, and ADC/ESP32-S3 communication board. Figure S3. (a) 12-lead spatial orientation (limb vs. precordial leads). (b) Representative precordial waveforms (V1–V6) from BioShield-12. Figure S4. Radar chart of normalized signal quality metrics (SNR, PLIR, skewness, kurtosis, BWI) for BioShield-12 vs. BIOPAC MP36. Figure S5. Spectral verification of PLIR and Wi-Fi interference immunity: (a) Welch PSD, (b) high-resolution FFT at 50 Hz, (c) high-frequency PSD (100–1000 Hz), (d) per-lead PLIR comparison. Figure S6. Normalized multi-metric heatmaps comparing BioShield-12 and BIOPAC across gain-independent quality indicators. Figure S7. Morphological comparison of 4 representative leads between BioShield-12 and BIOPAC MP36, acquired simultaneously. Figure S8. 3D wavelet scalograms of all 12 leads, showing time-frequency energy distribution (5–25 Hz) across QRS complexes. Figure S9. Respiratory rate comparison with and without BioShield-12 via ECG-derived respiration (EDR), showing comparable heart rate, breathing rate, and PSD. Table ST1. Lead calculation formulas relative to the manubrium reference (Mason–Likar limb positions). Table ST2. Signal integrity metrics (SNR, skewness, kurtosis) for one representative subject. Table ST3. DC and AC level metrics (Mean, Std, RMS) for one representative subject. Table ST4. Shape and frequency-domain measures (Ptp, BWI, PLIR) for one representative subject.
