# Supplementary material for: BioShield-12: A 3D-Printed Conformal Chest Shield for Wireless 12-Lead ECG Acquisition
Source: Sensors (Basel). 2026 Aug 4;26(15):4936. doi: 10.3390/s26154936 (PMC13468728; doi:10.3390/s26154936)
Supplement: Supplementary file 1 [file sensors-26-04936-s001.zip › sensors-4358151-supplementary.pdf]

Supplementary Materials

for: *BioShield-12: A 3D-Printed Conformal Chest Shield for Wireless 12-Lead ECG Acquisition*

Ahsan Naveed [1], Rida-e- Fatima [1], Zia Mohy Ud Din [1], Abdullah Al Aishan [2], Hedi Ammar Guesmi [3,*], and Jahan Zeb Gul [4,*].

1 *Air University; ahsan.naveed@au.edu.pk, ridafatima1401@gmail.com, drzia@au.edu.pk*

2 *King Khalid University; aalaishan@kku.edu.sa*

3 *Qassim University; h.guesmi@qu.edu.sa*

4 *Maynooth University; jahanzeb.gul@mu.ie*

* *Correspondence: h.guesmi@qu.edu.sa, jahanzeb.gul@mu.ie*

This document contains detailed hardware descriptions, signal processing analyses, and supporting data tables referenced in the main manuscript. S1 = electronic subsystem and PCB/wireless hardware; S2 = lead-derivation framework; S3 = extended signal-quality results; S4 = ECG-derived respiration.

Section S1: Electronic Subsystem and PCB/wireless hardware

Electrocardiography is a technique based on the recording of low-amplitude cardiac biopotentials (usually 0.5–5 mV on the body surface), which are susceptible to baseline drift, power-line interference, and motion artifact. The analog front-end (AFE) should therefore be capable of very high common-mode rejection, frequency band-limiting resolution to the diagnostic ECG bandwidth (0.05–100 Hz), and sufficient gain to fill the digitizer dynamic range. The adopted AFE architecture, shown in Figure S1, comprises a four-stage cascaded signal-conditioning chain. In the first stage, an INA129 instrumentation amplifier is used, chosen because of its high input impedance (≥10 GΩ) and common-mode rejection ratio (CMRR ≥120 dB), which are designed to reduce the effects of electrode polarization and common-mode interference at the electrode–skin interface.

After the pre-amplification stage, an active Twin-T 50-Hz tuned notch filter reduces power-line interference. The notch is followed by a 0.01 Hz high-pass filter for elimination of DC offset and low-frequency baseline wander resulting from respiration and motion, without affecting ST-segment and T-wave morphology. A gain stage of 100 V/V amplifies the conditioned ECG signal to the full ADC input range. A low-pass filter at 100 Hz establishes the upper bandwidth limit and acts as the anti-aliasing filter prior to digitization. Digitization was performed using a 24-bit ADS1256 delta-sigma ADC with VREF = 3.3 V (REFP–REFN).

The ADS1256 does not have dedicated biopotential features (such as right-leg drive and lead-off detection), the analog front-end chain compensates with pre-amplification at a high gain, and a regulated analog filter before the analog-to-digital converter (ADC). The single-point manubrium reference, coupled to the analog ground, is a common reference for each channel, which rejects common-mode signals. This is supported by experimentally measured power-line interference ratios (PLIR) of $1.0\text{-}3.2 \times 10^{-5}$, which shows that the power-line interference is reduced to levels that are insignificant for diagnosis.

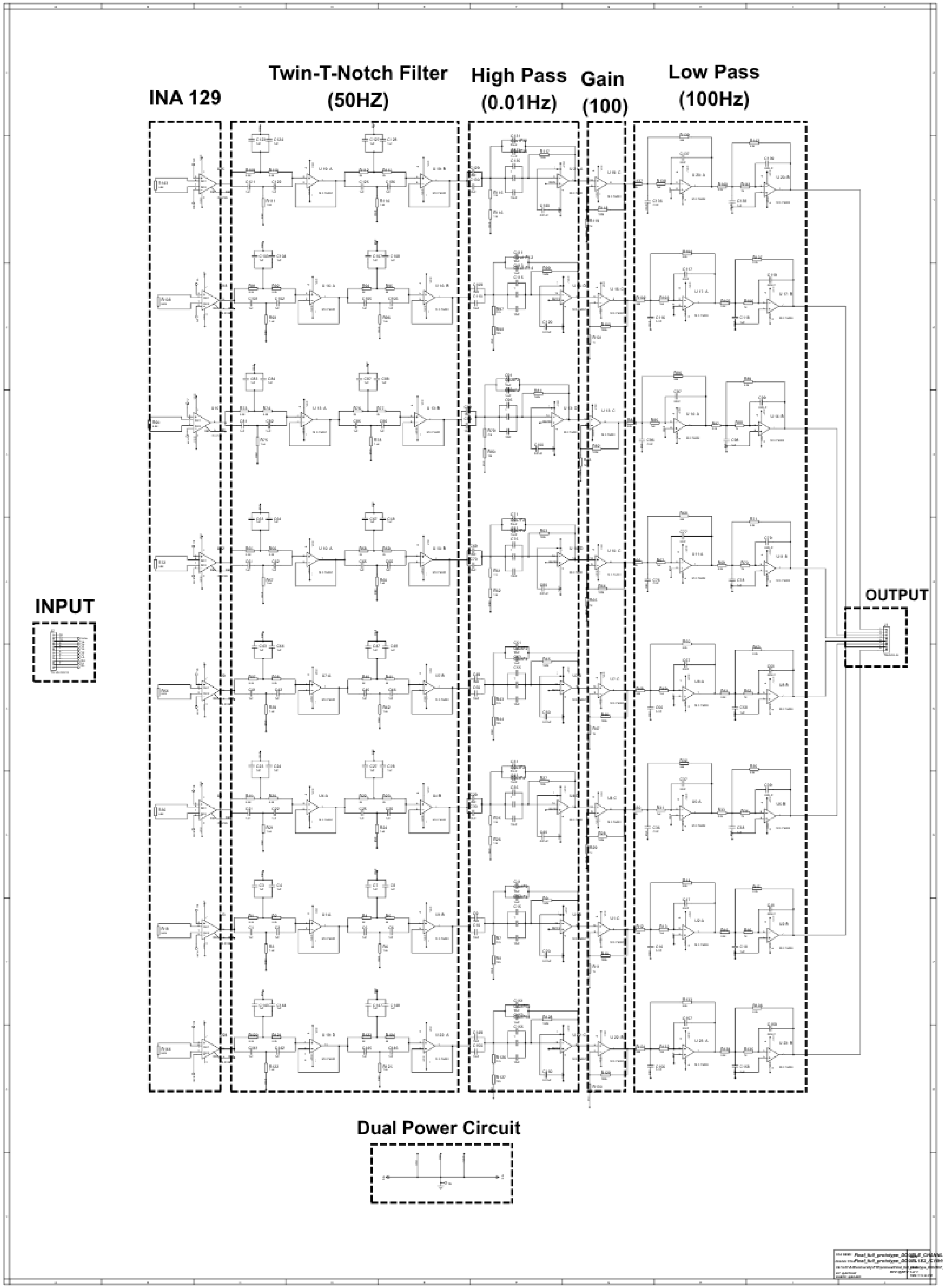

Figure S1. Schematic diagram of the 8-channel ECG acquisition front-end, showing input protection, INA129 instrumentation amplifier, Sallen–Key filter stages, and ADS1256 ADC interface. The nominal front-end voltage gain of 800 V/V is split into 8 V/V at the INA129 stage, followed by a 100 V/V post-filter gain stage.

PCB Stack Architecture

Printed Circuit Board Design and Architecture

In the design of a wearable device with high-quality signal processing, electromagnetic compatibility (EMC) immunity and noise immunity have been considered to enable high-grade signal fidelity. The vertical stack is a four-board structure that isolates the extremely sensitive analog signal-conditioning path from the high-speed digital processing and power management circuits, removing the possibility of capacitive coupling and ground loops. The continuous ground plane is star-grounded for return paths and to prevent ground potential variation. Guard rings surround the high-impedance nodes of the instrumentation amplifiers and analog signals are carried in differential pairs to the inputs of the ADC to improve common-mode rejection. The system is distributed across four vertically integrated PCBs, as illustrated in Figure S2, each dedicated to a specific functional domain:

Analog Acquisition Modules (Layers 1 & 2): Two identical 60 mm × 60 mm PCBs form the analog front-end. Each board independently conditions four ECG channels with precision INA129 instrumentation amplifiers and active filtering stages, ensuring channel-to-channel isolation and simplified signal routing for the 12-lead configuration.

Power Management Unit (Layer 3): A 70 mm × 78 mm board handles all power requirements. It incorporates a lithium-ion battery pack (2 cells) with a BMS (Model: HX-2S-JH10) providing overcharge, over-discharge, and short-circuit protection. An MT3608 DC-DC boost converter generates stable ±3.7 V analog rails and a 5 V digital rail, along with a low-noise reference voltage for consistent ADC biasing.

Digital Processing and Communication (Layer 4): The top layer (43 mm × 84 mm) houses the ADS1256 24-bit delta-sigma analog-to-digital converter (ADC) and ESP32-S3 microcontroller, providing high-resolution simultaneous digitization, real-time signal processing, and secure wireless transmission, electrically isolated from the analog layers. The electronic assembly is encased in a stiff enclosure (12 cm x 10 cm x 2 cm) embedded in the anterior side of the shield. The TPU shield flexes around the enclosure circumference, while the enclosure flexes little during normal breathing. Electrode cables are routed through strain-relief channels to absorb bending and avoid mechanical strain at the PCB connector or electrode-skin interface.

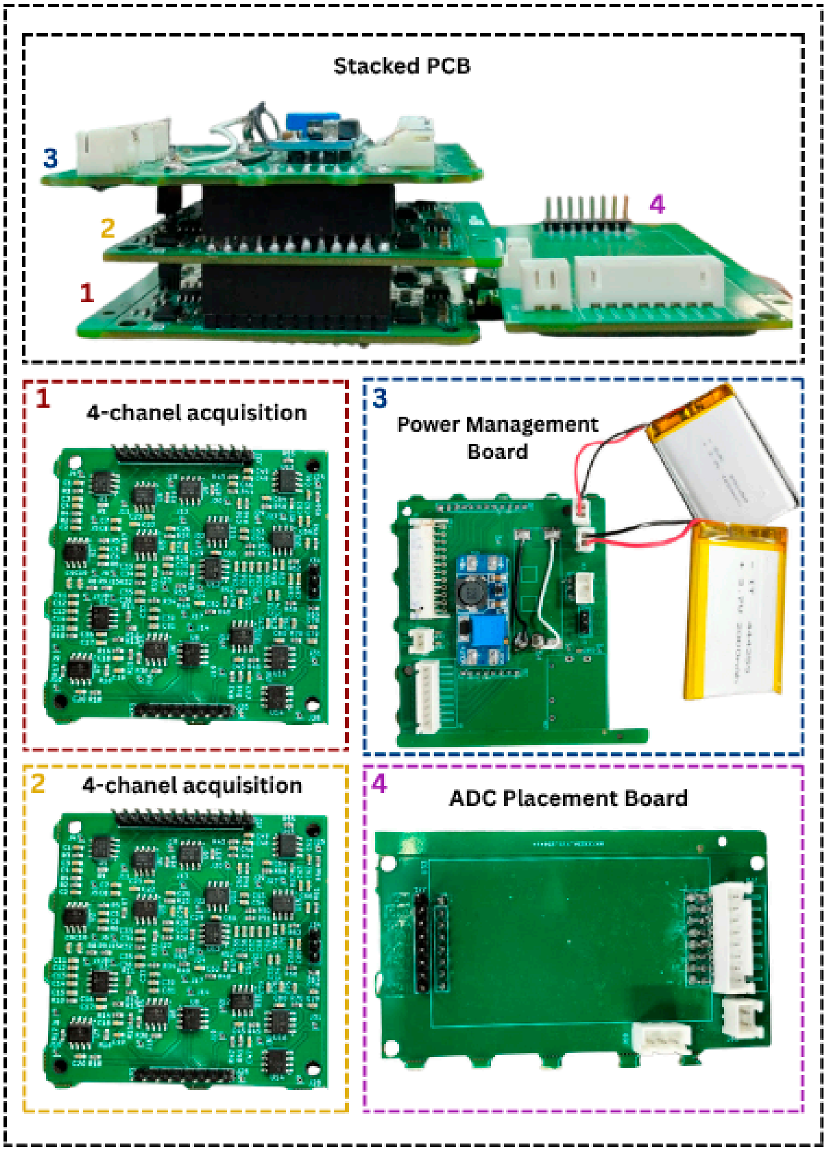

Figure S2. View of the four-board stacked PCB assembly. From top to bottom: power management and electrode I/O board; two 4-channel ECG acquisition boards; and the ADC/ESP32-S3 communication board.

Section S2: Lead Formation and Signal Derivation

Lead Formation and Signal Derivation Framework

The BioShield-12 lead formation scheme is tailored to the single-piece shield geometry: nine real physical sites (V1-V6, RA, LA, LL) are acquired with respect to a common manubrium sterni reference, and the 12-lead set is formed algebraically from the torso sites. This configuration differs from the clinical setup in two ways: (1) the limb electrode positions are based on the Mason-Likar modification ("torso-repositioned" limbs), which matches the fixed channels of the printed shield; and (2) the Wilson Central Terminal is replaced by the manubrium sterni as the unipolar reference, as is common to established wearable ECG lead systems (EASI, CM5, Fontaine) and the mechanical constraints of a wearable torso-only device.

Mathematical Derivation of ECG Leads

The acquisition architecture achieves the simplification of the analog front-end by transforming all the potentials into unipolar measurements relative to a common electrode as demonstrated in Table ST1. Precordial leads (V1-V6) are recorded relative to the manubrium sterni, which is electromechanically stable and has a low electromyographic (EMG) noise profile, eliminating hardware complexity to make the analog Wilson Central Terminal (WCT).

The manubrium acts as the common system reference, ensuring high-fidelity potential capture throughout the precordium:

$$Vn = ChestVn - ManubriumRef, \quad n = 1, \ldots, 6$$

The manubrium sterni was selected based on its established use in ECG systems (e.g., EASI, CM5, Fontaine) and its favorable properties: low EMG interference, mechanical stability, and consistent anatomical positioning. Wilson Central Terminal is unreliable in torso-repositioned mode (Mason–Likar), where limb potentials are not equally distributed (Gargiulo et al., 2015).

Table ST1. Lead Calculation Formulas. All potentials are measured relative to the manubrium reference. Limb leads use torso-repositioned (Mason–Likar) electrode positions.

LEAD	FORMULA
Lead I	*LA − RA*
Lead II	*Lead I + Lead III*
Lead III	*LL - LA*
aVR	*RA − (LA + LL) / 2*
aVL	*LA − (RA + LL) / 2*
aVF	*LL − (RA + LA) / 2*
V1	*ChestV1 − ManubriumRef*
V2	*ChestV2 − ManubriumRef*
V3	*ChestV3 − ManubriumRef*
V4	*ChestV4 − ManubriumRef*
V5	*ChestV5 − ManubriumRef*
V6	*ChestV6 − ManubriumRef*

Real-Time Signal Digitization and Lead Computation

Biopotential signals at the physical locations (V1-6, RA, LA, LL) from the manubrium are simultaneously acquired by the ADS1256 ADC at a sampling rate of 1000 Hz with an ESP32-S3 microcontroller. After digitization, the microcontroller removes DC offset and uses a low-pass filter to filter out high-frequency noise (beyond the diagnostic ECG frequency range). The precordial leads (V1 - V6) are directly sampled and do not need further calculations. Limb leads are computed: Lead I and Lead III from RA, LA and LL as per Table ST1; Lead II from Lead I and Lead III using Einthoven's law; and augmented leads (aVR, aVL, aVF) using Goldberger's formulas. This process guarantees all 12 leads of the ECG are reconstructed for wireless broadcasting and real-time display, as depicted in Figure S3.

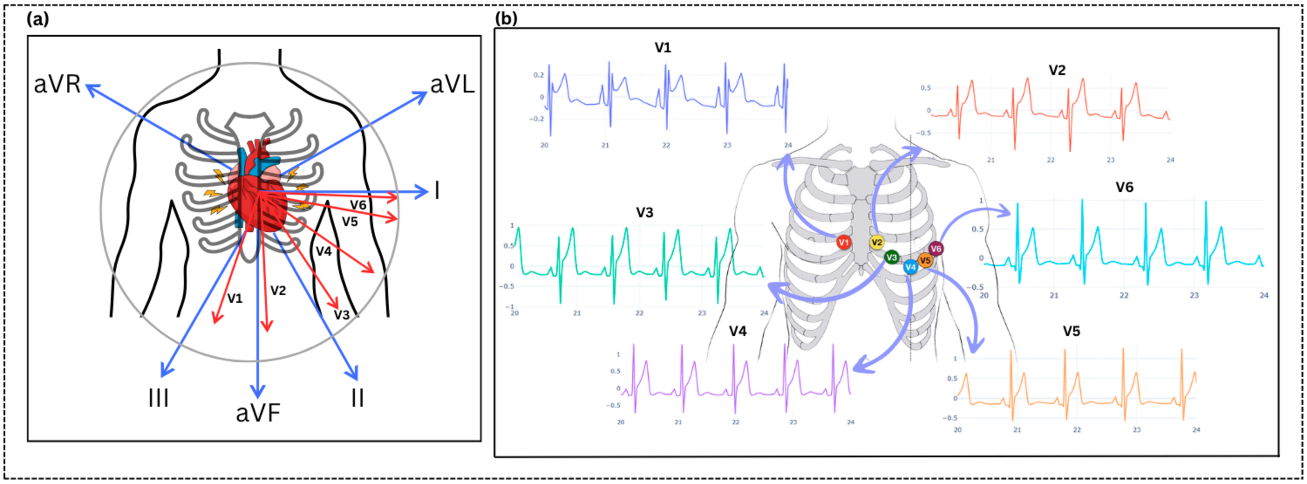

Figure S3. (a) Schematic representation of the 12-lead ECG spatial orientations: limb leads (blue) capture frontal-plane electrical activity, while precordial leads (red) capture horizontal-plane activity. (b) Representative waveforms acquired from precordial leads V1–V6 using the BioShield-12 system.

Section S3: Extended Signal Quality Results

Quantitative Performance Analysis

To facilitate a fair comparison of BioShield-12 front-end performance that is not affected by the 800 V/V gain, we calculated the performance metrics using normalized units, as shown in Figure S4. The most significant result was the noise immunity comparable to the industry standard, with PLIR between 1.0 and 3.2×10^{-5}, allowing effective suppression of the 50/60 Hz noise. And consistent skewness ensured preservation of the natural asymmetry of the ECG. Kurtosis analysis showed BioShield-12 generated moderate kurtosis, representative of actual physiological morphology, while the reference system generated higher kurtosis (7–16) in some leads, reflecting internal processing that artificially sharpens peaks. BioShield-12, therefore, provides a more faithful replica of the native physiological signal.

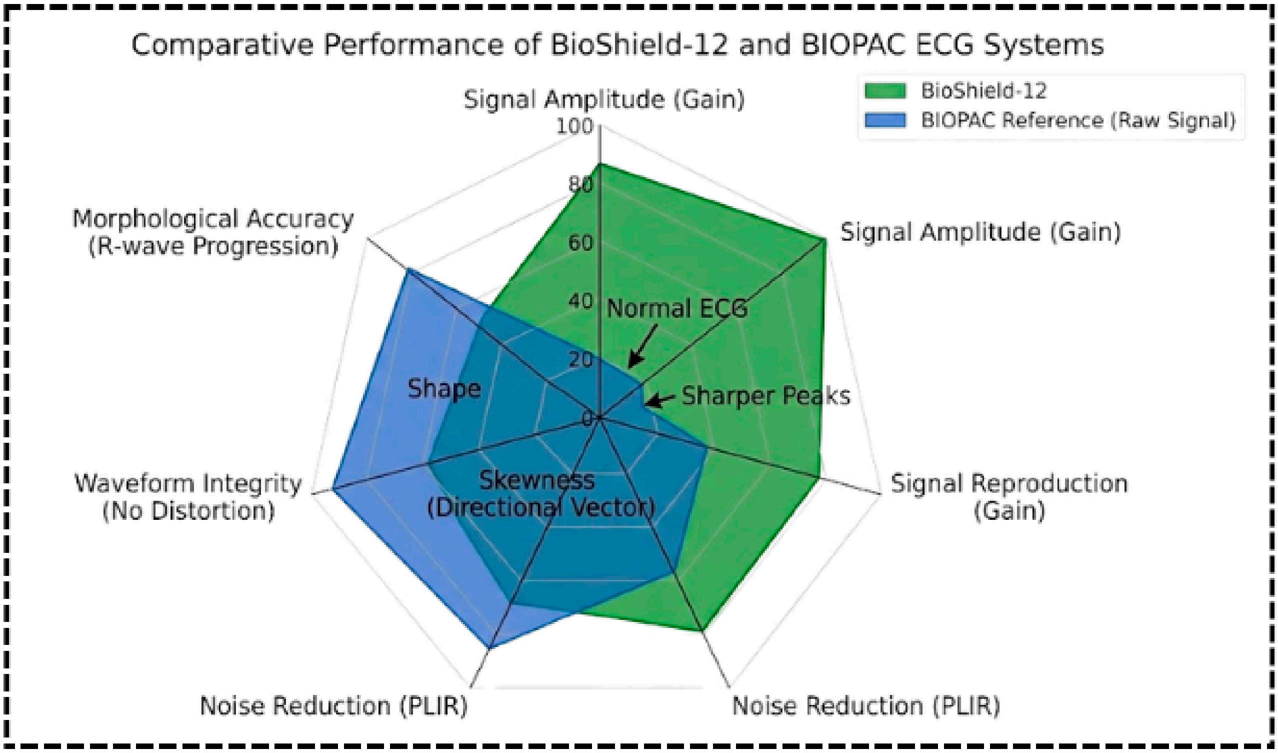

Figure S4. Radar chart comparing BioShield-12 and BIOPAC MP36 across normalized signal quality metrics (SNR, PLIR, skewness, kurtosis, bandwidth index). Amplitude-based metrics (RMS, PtP) are excluded as they reflect system gain rather than signal quality.

Spectral Verification of PLIR and Wi-Fi Interference Immunity

To provide spectral evidence supporting the reported PLIR values and address potential interference from the ESP32-S3 Wi-Fi subsystem, spectral noise analysis was performed on representative BioShield-12 recordings (Figure S5). Welch PSD analysis (0–1000 Hz, 8192-point Hann window, 50% overlap) shows ECG energy concentrated within the 0.5–40 Hz diagnostic band, with the 50 Hz mains component at the noise floor (~4.55×10^{-6} mV^2/Hz). A high-resolution FFT confirms the residual 50 Hz amplitude is approximately 1.52 μV (≈72 dB below the dominant QRS amplitude), yielding PLIR consistent with the $1.0–3.2 \times 10^{-5}$ range reported in Table ST4. The 100–1000 Hz region shows a flat noise floor without discrete peaks, confirming the absence of structured electromagnetic interference. The ESP32-S3 Wi-Fi radio operates at 2.4 GHz, far above the 1 kHz Nyquist limit, so any RF coupling manifests only as broadband noise rather than discrete spectral components in the ECG band.

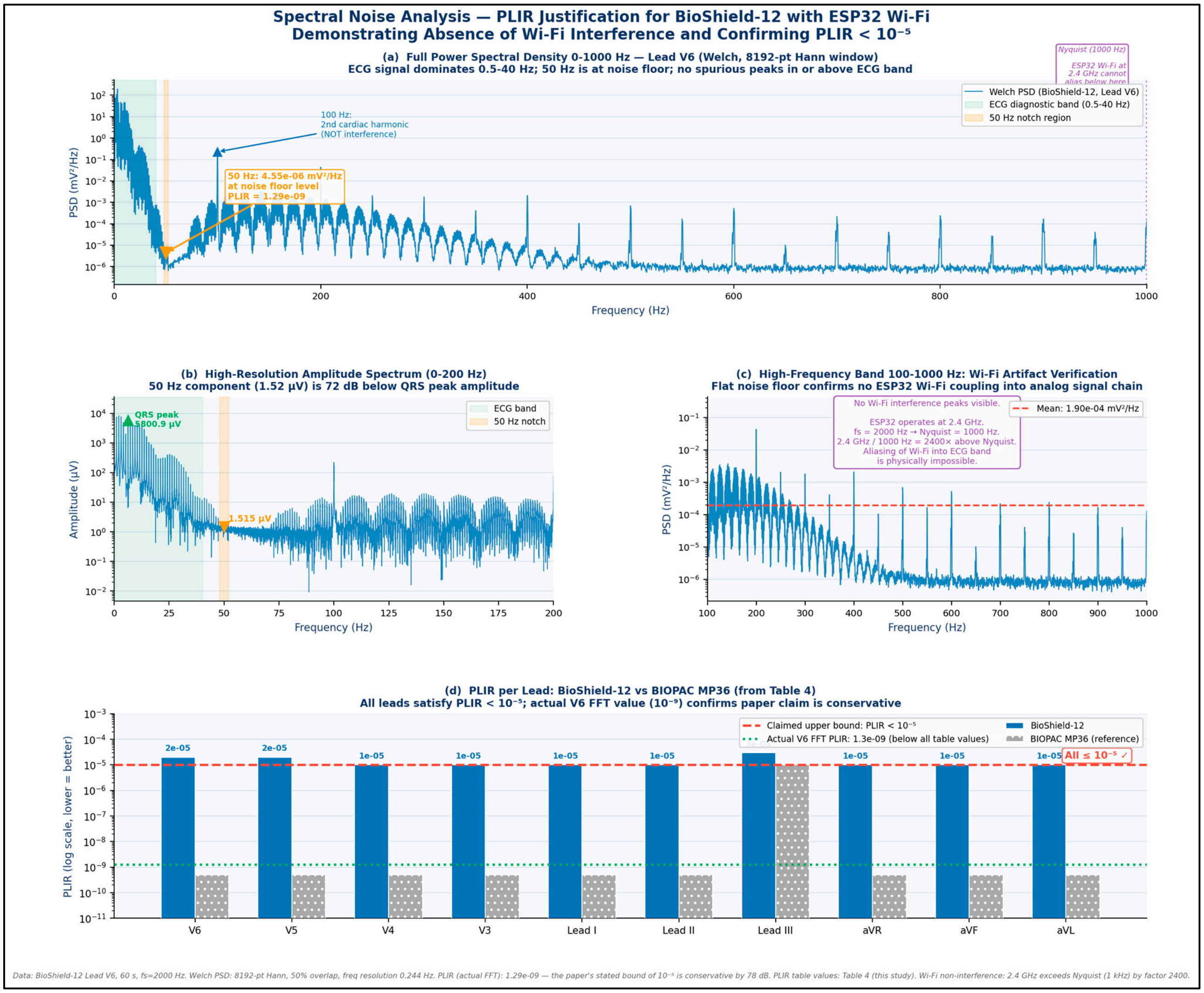

Figure S5. Spectral analysis supporting PLIR and interference immunity. (a) Welch PSD (0–1000 Hz) showing ECG energy confined to the 0.5–40 Hz band with the 50 Hz mains component at the noise floor. (b) High-resolution FFT (0–200 Hz) showing a 50 Hz amplitude of 1.52 µV (≈72 dB below the QRS peak). (c) High-frequency PSD (100–1000 Hz) showing a flat noise floor with no Wi-Fi interference peaks. (d) Per-lead PLIR comparison with BIOPAC MP36, confirming all BioShield-12 leads satisfy PLIR $\leq 3.2 \times 10^{-5}$.

Multi-Metric Performance Evaluation

High system fidelity at complementary dimensions was verified with multi-metric testing with normalized and gain-independent parameters, as shown in Figure S6. Normalized values of kurtosis (0.94-0.96) ensured that the shape of the ECG waveforms was not distorted artificially. The BWI (≈0.99) corresponded to uniform, even frequency response in all leads with uniform capture of diagnostic frequency content. Normalized PLIR (≈0.94) showed that suppression of 50/60 Hz mains interference had been successfully achieved. These results confirm the finding that the custom analog front-end can obtain signal integrity on the level of research-grade instrumentation.

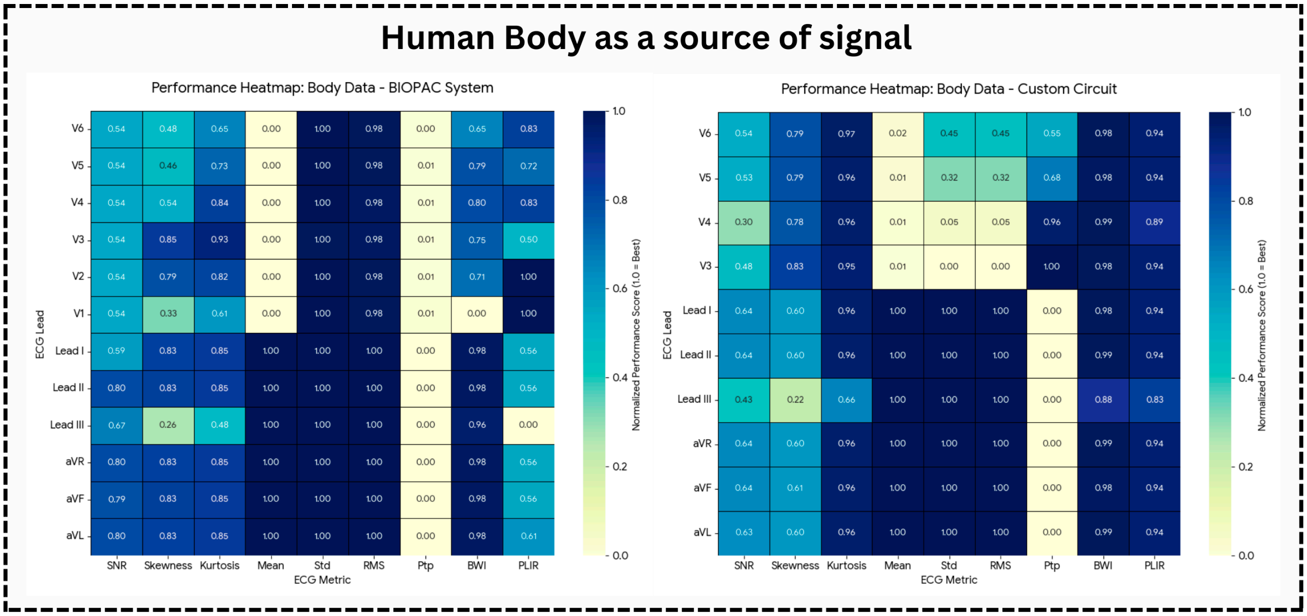

Figure S6. Normalized multi-metric heatmaps comparing BioShield-12 and BIOPAC performance across gain-independent quality indicators. Higher normalized values (closer to 1.0) indicate better performance for SNR, BWI, and PLIR metrics.

Morphological Comparison

There was high agreement in ECG recordings obtained by BioShield-12 and BIOPAC MP36 in real-time on the healthy subjects, as illustrated in Figure S7. Signals exhibited excellent time resolution and maintained all the primary morphological features, including P waves, QRS complexes, and T waves, with stable baselines and no apparent distortion or drift. The cross-correlation analysis, results were high correlation coefficients among all the subjects, which validated the reliability of BioShield-12 in the reproduction of diagnostic ECG waveform structure.

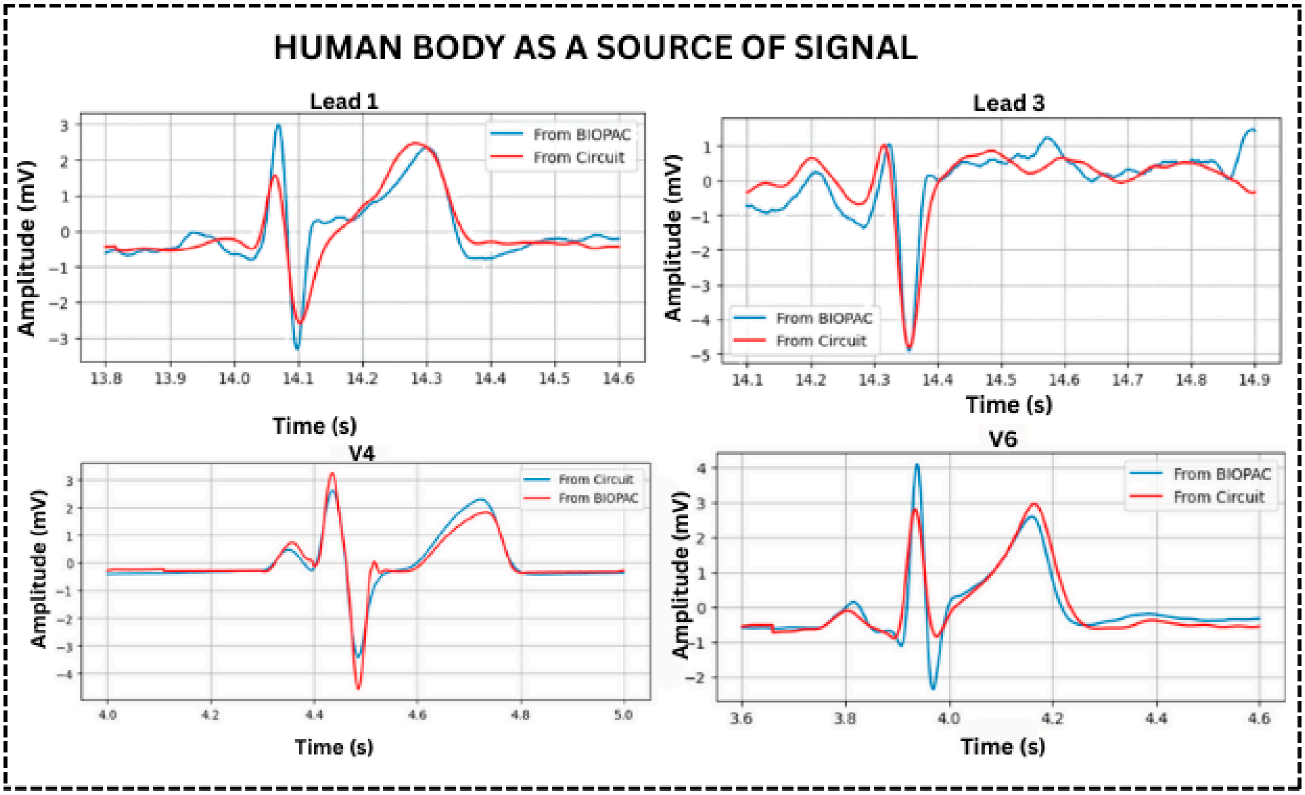

Figure S7. Human subject-based morphological comparison showing 4 representative leads (out of 12) comparing BioShield-12 and BIOPAC MP36 waveforms acquired simultaneously. Signals demonstrate comparable P waves, QRS complexes, and T waves, with consistent baselines and no observable distortion or drift.

Time-Frequency Analysis

Figure S8 shows time-frequency analysis of the BioShield-12 system using 3D wavelet scalograms which indicate that signal energy is concentrated in 5-25 Hz band across QRS complexes, as expected of physiological ECG spectral properties. Scalograms show physiological values V1-V6 and stable limb lead profiles and no aliasing, nonlinear distortion or spurious high-frequency content are observed, which validate high-fidelity signal capture of all 12 leads.

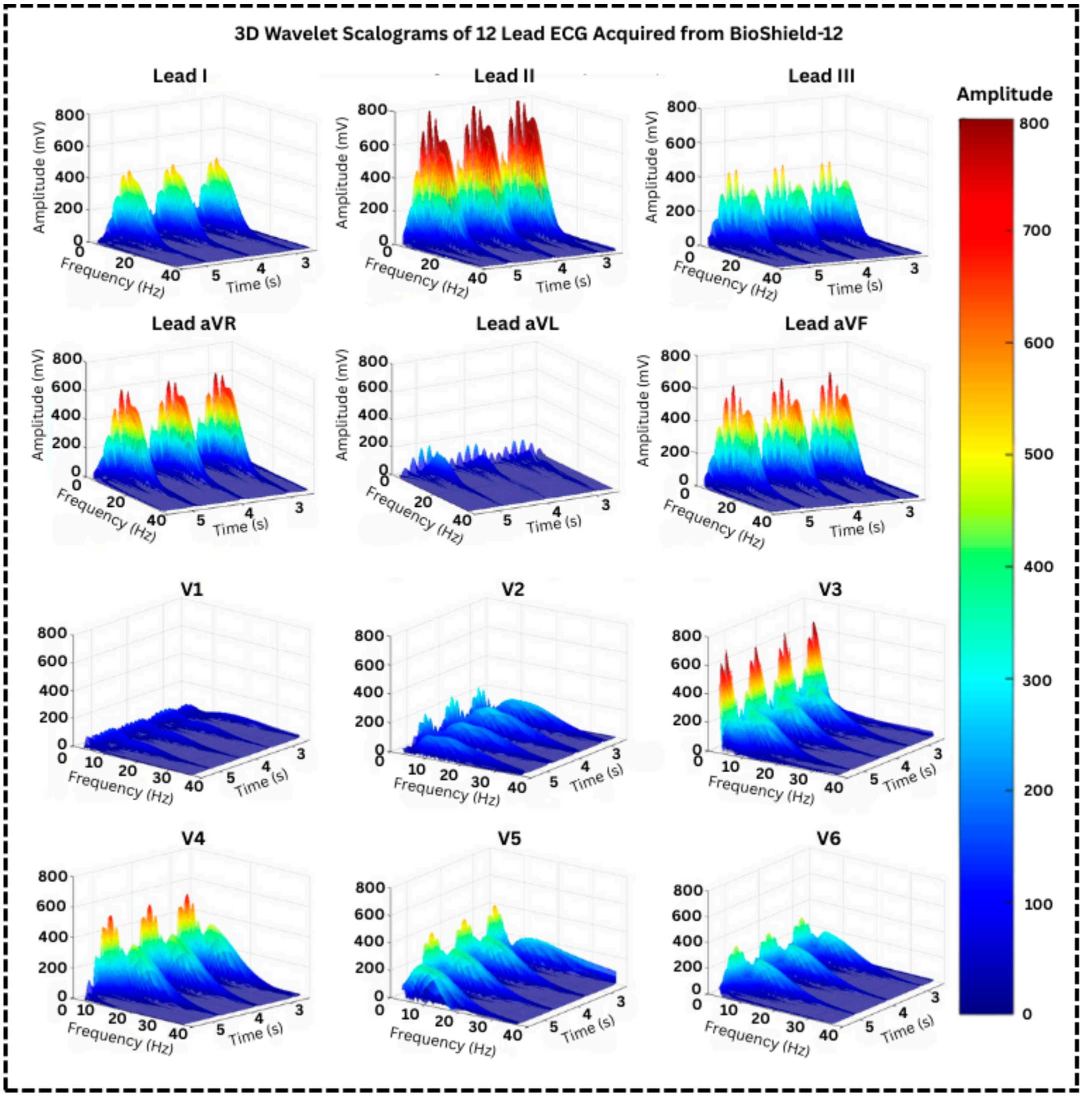

Figure S8. 3D wavelet scalograms of all 12 ECG leads of the BioShield-12 system, showing time-frequency distribution and amplitude of cardiac activity. Energy is concentrated at 5–25 Hz across QRS complexes, with physiological trends from V1–V6 and stable limb lead profiles, confirming high-fidelity signal capture.

Section S4: ECG-Derived Respiration Analysis

ECG-Derived Respiration: Validation of Unimpeded Breathing With and Without BioShield-12

ECG-Derived Respiration (EDR) analysis was conducted to ensure BioShield-12 does not hinder breathing when worn. EDR leverages respiratory changes to the ECG in: (i) cyclical amplitude variation of the R-wave, due to cardiac electrical axis rotation and thoracic impedance, and (ii) low-frequency baseline wander (0.1-0.5 Hz), due to changes in electrode impedance and respiration-induced thoracic motion. Therefore, respiratory signals can be extracted from the ECG, obviating the need for respiratory leads. EDR was implemented on ECGs from standard leads. In the with-shield case, a 60-second recording in supine rest was considered (Figure S9a,c,e). R-peaks were detected from the ECG bandpass filtered (0.5-100 Hz), showing stable heart activity with regular RR intervals and no body motion artifacts. Baseline wander was obtained by down-sampling to 50 Hz, bandpass filtering (0.1-0.5 Hz), and taking samples at R-peak locations. When added to the R-amplitude series, smoothed at 0.8 Hz and resampled at 10 Hz, the resulting respiratory waveform showed regular inspiration-expiration cycles with a breathing rate in the normal range for rest (12-20 breaths/min). Power spectral density (PSD) analysis confirmed the major energy of respiration in the

physiological range (0.1-0.5 Hz) with no artifacts. Applying the same EDR method to a matched unshielded recording from the same subject and session (Figure S9b,d,e) yielded a difference in breathing rate of just 0.8 breaths/min, within the normal variability of breathing rates and not clinically relevant. The summary plot (Figure S9f) shows identical heart rate, breathing rate and number of detected breath cycles in the shielded and unshielded cases. Owing to the high front-end gain (800 V/V), R-wave amplitudes were close to saturation for the ADC, precluding a significant contribution from amplitude-based respiratory modulation; the respiratory signal was thus derived primarily from baseline wander, a proven and robust EDR technique with high front-end gain. These results also suggest the mechanical and electrical stability of BioShield-12. During continuous recording with active breathing, no signal dropouts, impedance transients or bursts of motion artifact were detected. The stable cardiac cycle confirms the stability of electrode-skin contact despite chest movement.

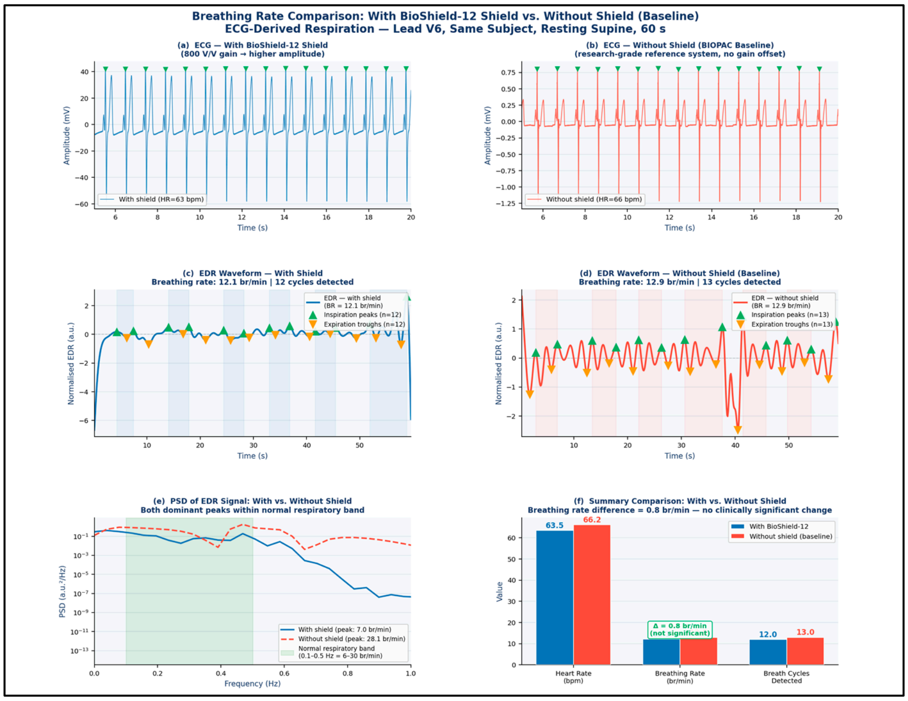

Figure S9. Respiratory rate with and without BioShield-12 (baseline) Data obtained from the same subject with same resting supine posture (60 s); one lead (V6) is shown. (a-b) ECG segments display similar heart activity. (c-d) EDRs show comparable breathing rates (12.1 vs. 12.9 breaths/min). (e) PSD shows the main spectral power in the physiological breathing range (0.1-0.5 Hz). (f) Key metrics are similar, with a 0.8 breaths/min difference (not clinically significant), showing no effect of the shield on breathing mechanics.

Supplementary Tables

Table ST2. Signal integrity metrics (SNR, skewness, kurtosis) for one representative subject. Values are dimensionless or in dB and are independent of system gain. For multi-subject mean ± SD (n = 20) with 95% CI, see Table 4 in the main manuscript.

Signal	SNR (BioShield, dB)	SNR (BIOPAC, dB)	Skewness (BioShield)	Skewness (BIOPAC)	Kurtosis (BioShield)	Kurtosis (BIOPAC)
V1	17.50	19.80	-1.52	1.74	4.15	9.60
V2	17.80	19.80	-1.49	1.71	4.20	9.85
V3	17.75	19.69	-1.48	0.55	4.09	4.84
V4	12.47	19.69	-1.28	1.68	3.54	7.40
V5	19.13	19.69	-1.58	1.95	4.27	10.33
V6	19.44	19.69	-1.63	1.87	4.51	12.30
Lead I	22.44	20.96	1.45	-0.61	4.02	6.98
Lead II	22.54	27.05	1.44	-0.60	4.01	6.99

Lead III	16.41	23.44	-2.82	2.67	12.20	16.78
aVR	22.50	27.00	-1.45	0.61	1.42	6.99
aVF	22.57	26.86	1.42	-0.60	1.42	6.99
aVL	22.30	27.13	1.44	-0.61	1.44	6.97

Table ST3. DC and AC level metrics (Mean, Std, RMS) for one representative subject. BioShield-12 values reflect the 800 V/V front-end gain and are not directly comparable in magnitude with BIOPAC.

Signal	Mean (BioShield)	Mean (BIOPAC)	Std (BioShield)	Std (BIOPAC)	RMS (BioShield)	RMS (BIOPAC)
V1	−0.00000600	−0.00100	0.000280	0.000290	0.000280	0.001010
V2	−0.00000590	−0.00100	0.000320	0.000310	0.000320	0.001020
V3	−0.00000600	−0.00100	0.000392	0.000384	0.000392	0.001070
V4	−0.00000335	−0.00100	0.000377	0.000325	0.000377	0.001050
V5	−0.00000600	−0.00100	0.000267	0.000276	0.000267	0.001036
V6	−0.00000595	−0.00100	0.000209	0.000225	0.000210	0.001024
Lead I	0.00001	0.00002	0.0000018	0.000179	0.0000018	0.000179
Lead II	−0.00002	0.00001	0.0000018	0.000179	0.0000018	0.000179
Lead III	0.00001	−0.00001	0.0000011	0.000121	0.0000011	0.000121
aVR	−0.00002	0.00001	0.0000018	0.000179	0.0000018	0.000179
aVF	0.00002	−0.00001	0.00000185	0.000179	0.00000185	0.000179
aVL	0.00001	0.00001	0.0000018	0.000179	0.0000018	0.000179

Table ST4. Shape and frequency-domain measures (Ptp, BWI, PLIR) of a single representative. V tells BioShield-12 (800 V/V gain) and mV tells BIOPAC; not directly comparable systems. The BWI is the ratio of power in the 0.05-100 Hz diagnostic band, to total acquisition bandwidth power, on a non-dimensional basis. Direct comparison of the two systems is done by comparing the values of PLIR as $\times 10^{-5}$.

Signal	Ptp (BioShield)	Ptp (BIOPAC)	BWI (BioShield)	BWI (BIOPAC)	PLIR (BioShield, $\times 10^{-5}$)	PLIR (BIOPAC, $\times 10^{-5}$)
V1	0.2150	2.620	0.0510	0.3650	3.2	<0.1
V2	0.2290	2.730	0.0540	0.3720	3.0	<0.1
V3	0.4510	3.896	0.0447	0.2888	1.0	<0.1
V4	0.4312	3.281	0.2045	0.2351	1.0	<0.1
V5	0.3053	3.005	0.0284	0.2417	2.0	<0.1
V6	0.2481	2.842	0.0583	0.3881	2.0	<0.1
Lead I	0.00178	1.834	0.0394	0.0449	1.0	<0.1
Lead II	0.00177	1.831	0.0383	0.0444	1.0	<0.1
Lead III	0.00165	1.508	0.1512	0.0655	3.0	1.0
aVR	0.00178	1.832	0.0386	0.0446	1.0	<0.1
aVF	0.00179	1.837	0.0416	0.0454	1.0	<0.1
aVL	0.00176	1.828	0.0384	0.0439	1.0	<0.1
